# The global burden of vertebral fractures caused by falls among individuals aged 55 and older, 1990 to 2021

**DOI:** 10.1371/journal.pone.0318494

**Published:** 2025-04-08

**Authors:** Yao-Kan Zhang, Jia-Xuan Wang, Yi-Zhou Ge, Ze-Bin Wang, Zhi-Guo Zhang, Zhong-Wei Zhang, Feng Chang

**Affiliations:** 1 The Orthopedic Department of Shanxi Provincial People’s Hospital, Shanxi Medical University, Taiyuan, China; 2 Fifth Clinical Medical College, Shanxi Medical University, Taiyuan, China; 3 First Clinical Medical College, Changzhi Medical College, Changzhi, China; Facultad Latinoamericana de Ciencias Sociales Mexico, MEXICO

## Abstract

**Purpose:**

This study provides a comprehensive analysis of the global incidence, prevalence, and years lived with disability (YLDs) attributable to vertebral fractures from falls among individuals aged 55 and older between 1990 and 2021, with trends further delineated by gender, geographic region, and socio-demographic index (SDI).

**Methods:**

This study utilized data from the 2021 Global Burden of Disease (GBD) study, focusing on trend changes and stratified characteristics of the burden of vertebral fractures caused by falls among individuals aged 55 and older.

**Results:**

In 2021, there were approximately 2.02 million new cases of vertebral fractures due to falls among individuals aged 55 and older globally, with 2.70 million prevalent cases and 264,211 YLDs. The age-standardized incidence rates (ASIR) in 2021 was 140.77 per 100,000, showing an increase compared to 1990 (average annual percent change [AAPC]: 0.27; 95% confidence interval [CI]: 0.23 to 0.30), while the age-standardized prevalence rates (ASPR) and age-standardized years lived with disability rates (ASYR) exhibited a downward trend. Female patients had higher indicators than male patients, but the burden on male patients was increasing. The ASIR, ASPR, and ASYR in high SDI regions were positively correlated with SDI. High-income and densely populated regions and countries bore the greatest burden. Predictive analysis showed that the global burden of vertebral fractures will further increase between 2022 and 2035.

**Conclusions:**

From 1990 to 2021, the burden of vertebral fractures due to falls among individuals aged 55 and older showed an upward trend. The burden on males may have been underestimated, and particular attention is required for high SDI regions, high-income areas like North America and Western Europe, as well as densely populated countries. With the aging population, vertebral fractures caused by falls require continued attention.

## 1 Introduction

Vertebral fractures can arise from trauma at various energy levels [1]. In elderly individuals, even minor trauma can lead to vertebral fractures [2]. This heightened susceptibility is typically attributed to underlying osteoporosis [3]. Osteoporosis is a global health issue. Even in countries like the United States, where there is extensive medication coverage, the assessment and treatment of osteoporosis remain inadequate [4]. Vertebral compression fractures are the common type of osteoporotic fractures, typically resulting from low-impact injuries [5, 6]. In the elderly, vertebral fractures may result in recurrent fractures, intensified pain, and elevated mortality [7, 8], while placing substantial social, healthcare, and economic burdens [9–11].

Due to degenerative changes and a rise in underlying disease, elderly individuals often experience prolonged recovery following fractures. The impact of identical fractures is typically far more severe in older adults than younger counterparts. With the global rise in aging populations, addressing elderly health concerns is poised to be a critical challenge for future healthcare systems. Previous studies have evaluated the epidemiology of vertebral fractures in the general global population [12], but recent assessments of the epidemiology of vertebral fractures specifically caused by factors in the elderly population are lacking. The epidemiology of vertebral fractures among the elderly reveals distinct regional variations [13, 14].

Falls are a predominant cause of fractures among the elderly, significantly contributing to the burden of geriatric fractures [15, 16]. This may be due to reduced activity, decline in bodily functions, or dementia [17]. Complications related to falls in the elderly population can lead to severe consequences [18]. Additionally, it imposes a dramatic financial burden on society [19], hindering the implementation of healthy aging tactics. The guidelines from the American Geriatrics Society and the British Geriatrics Society recommend annual fall risk screening for patients aged 65 years or older [20].

The Global Burden of Disease (GBD) study utilizes publicly available data and standardized methodologies to provide a comprehensive perspective on the burden of diseases, injuries, and risk factors [21]. This study not only analyzes global incidence, prevalence, and years lived with disability (YLDs) due to vertebral fractures caused by falls among individuals aged 55 and older across 204 countries and regions from 1990 to 2021, along with age-standardized incidence rates (ASIR), age-standardized prevalence rates (ASPR), and age-standardized years lived with disability rates (ASYR), but also provides stratified trends by gender, region, country, and Socio-Demographic Index (SDI). The findings present an updated, in-depth perspective for global researchers and high-risk populations. These insights will allow for better implementation of healthcare practices, resource allocation, and policy-making.

## 2 Methods

### 2.1 Data sources

The analysis was conducted using data from GBD 2021, which comprehensively evaluated the incidence, prevalence, and YLDs of 371 diseases and injuries, along with 88 risk factors, across 204 countries and regions during this period. Data on vertebral fractures were sourced from records of various countries [21,22]. The data were subsequently modeled to estimate the incidence, prevalence, and YLDs of vertebral fractures. The burden of disease refers to the overall socioeconomic and health impact, leading to poor health, disability, and premature death.

The study classified injuries into two aspects: cause and nature. Injury causes encompass include road injuries, falls, interpersonal violence, and other factors that directly affect the body. GBD categorized injury causes across four tiers, with Level 1 being the broadest and Level 4 offering the most granular detail [23]. Comprehensive details on injury data sources have been published previously [24]. Falls at Level 3 were chosen as the primary injury cause. The nature of injury refers to the physical outcomes of the cause, such vertebral fractures. A single injury cause can lead to multiple injury types. Vertebral fractures are considered as the nature of the injury, rather than the cause [25]. Vertebral fractures were defined in the GBD study using the International Classification of Diseases (ICD-9 and ICD-10) codes. The GBD study methodology aggregates data from various sources and does not explicitly specify ICD-9 or ICD-10 codes for vertebral fractures. Fracture data were divided into nine age groups: 55-59, 60-64, 65-69, 70-74, 75-79, 80-84, 85-90, 90-94, and 95 + years. Moreover, not all vertebral fractures in the GBD study were osteoporotic fractures. Previous research has indicated that in patients aged 55 and older, vertebral fractures were primarily caused by osteoporosis.

SDI represents a composite metric reflecting the socioeconomic conditions influencing health outcomes across various regions. SDI is calculated using the geometric mean of three key indicators: total fertility rate under 25 years (TFR < 25), average years of education for those aged 15 and older (EDU15+), and lag-distributed income (LDI) per capita. In the GBD 2021 study, SDI values range from 0 to 1, with higher values indicating better socio-economic conditions and improved health outcomes. Based on 2021 SDI, regions are divided into five quintiles: low (0-0.466), low-middle (0.466-0.619), middle (0.619-0.712), high-middle (0.712-0.810), and high (0.810-1) [21].

### 2.2 Statistical analysis

The absolute values of incidence, prevalence, and YLDs were calculated by summing the corresponding values of all relevant 5-year age groups. The estimates for vertebral fractures attributable to falls in individuals aged 55 and older were age-standardized using direct age-standardization. The goal of age standardization is to account for variations in age distribution across populations. ASIR, ASPR, and ASYR were calculated per 100,000 population. Joinpoint regression models were used to analyze the time trends of the burden of vertebral fractures caused by falls in individuals aged 55 and older from 1990 to 2021. The Bayesian age-period-cohort (BAPC) model was employed to predict further the age-standardized rates (ASR) and number of vertebral fractures caused by falls among individuals aged 55 and older for the above indicators from 2022 to 2035. The number of vertebral fractures and ASR per 100,000 population were provided with 95% uncertainty intervals (UI). Spearman correlation analysis assessed the relationship between ASIR, ASPR, ASYR, and SDI. Time trends were assessed using the Joinpoint software (version 5.0.2). The average annual percent change (AAPC) and its 95% confidence interval (CI) were calculated using a linear regression model. A negative upper limit of the 95% CI for the AAPC indicated a downward trend, while a positive lower limit indicated an upward trend. All statistical analyses and plots were conducted using R statistical software (version 4.3.3). A two-sided p-value of < 0.05 was considered statistically significant.

## 3 Results

### 3.1 Global burden trends

**Fig 1:**
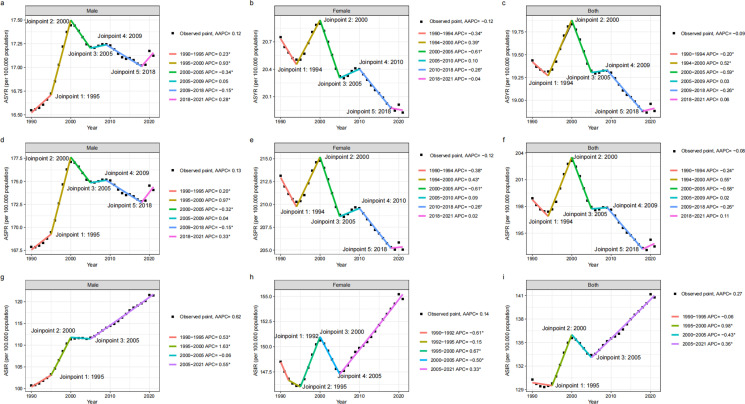
Joinpoint regression analysis in ASYR, ASPR, and ASIR of global vertebral fractures due to falls among individuals aged 55 and older from 1990 to 2021. Notes: From 1990 to 2021, the ASYR of vertebral fractures among individuals aged 55 and above globally, for males (a), females (b), and the total population (c). From 1990 to 2021, the ASPR of vertebral fractures among individuals aged 55 and above globally, for males (d), females (e), and the total population (f). From 1990 to 2021, the ASIR of vertebral fractures among individuals aged 55 and above globally, for males (g), females (h), and the total population (i). AAPC, average annual percent change; APC, annual percentage change; YLDs, years lived with disability; ASYR, age-standardized years lived with disability rate; ASPR, age-standardized prevalence rate; ASIR, age-standardized incidence rate.

In 2021, approximately 2.02 million incident cases of vertebral fractures caused by falls occurred globally among individuals aged 55 and older, an increase from 795,666 cases in 1990. An estimated 2.70 million prevalent cases of vertebral fractures due to falls were recorded globally in 2021, compared to 1.12 million cases in 1990. In 2021, among patients aged 55 and above worldwide, vertebral fractures caused by falls resulted in 264,211 YLDs, showing an upward trend compared to 1990. The ASIR for vertebral fractures caused by falls in individuals aged 55 and older was 140.77 per 100,000 in 2021, compared to 130.29 per 100,000 in 1990 (AAPC: 0.27; 95% CI: 0.23 to 0.30). The ASPR for vertebral fractures among individuals aged 55 and above was 193.50 per 100,000 in 2021, decreasing from 1990 (AAPC: -0.08; 95% CI: -0.12 to -0.04). Over the same period, the ASYR decreased from 19.44 to 18.88 per 100,000 (Table 1) (AAPC: -0.09; 95% CI: -0.13 to -0.05). Analysis revealed significant inflection points in ASIR in 1995, 2000, and 2005. The ASIR declined from 1990 to 1995 (APC =  -0.06, 95% CI: -0.16 to 0.05, P =  0.267), rose between 1995 and 2000 (APC =  0.98, 95% CI: 0.83 to 1.14, P <  0.001), fell again between 2000 and 2005 (APC =  -0.43, 95% CI: -0.58 to -0.28, P <  0.001), and continued rising from 2005 to 2021 (APC =  0.36, 95% CI: 0.34 to 0.38, P <  0.001) (Fig 1(i)). Significant ASPR inflection points were observed in 1994, 2000, 2005, 2009, and 2018. ASPR decreased from 1990 to 1994 (APC =  -0.24, 95% CI: -0.36 to -0.11, P =  0.001), increased between 1994 and 2000 (APC =  0.55, 95% CI: 0.47 to 0.64, P <  0.001), fell again between 2000 and 2005 (APC =  -0.58, 95% CI: -0.70 to -0.45, P <  0.001), and then rose between 2005 and 2009 (APC =  0.02, 95% CI: -0.18 to 0.22, P =  0.843). ASPR decreased slowly between 2009 and 2018 (APC =  -0.26, 95% CI: -0.31 to -0.22, P <  0.001), with a slight increase between 2018 and 2021 (APC =  0.11, 95% CI: -0.09 to 0.31, P =  0.271) (Fig 1(f)). The ASYR of vertebral fractures caused by falls in individuals aged 55 and older followed a similar pattern (Fig 1(c)).

**Table 1:** Incidence, prevalence, and YLDs of vertebral fractures caused by falls among elderly people in 1990 and 2021, and AAPC from 1990 to 2021, by global, sex, SDI quintile, and regional levels.

Location	Incidence					Prevalence					YLDs				
	Incident cases(1990)	ASIR(1990)	Incident cases(2021)	ASIR(2021)	AAPC 1990–2021	Prevalent cases(1990)	ASPR(1990)	Prevalent cases(2021)	ASPR(2021)	AAPC 1990–2021	YLDs(1990)	ASYR(1990)	YLDs (2021)	ASYR(2021)	AAPC 1990–2021
Global	795666(463397 to 1254757)	130.29(75.73 to 205.36)	2016676(1195228 to 3128813)	140.77(83.44 to 218.27)	0.27(0.23 to 0.30)	1117063(896116 to 1404065)	198.90(158.58 to 252.21)	2698265(2150309 to 3403083)	193.50(153.92 to 244.58)	-0.08(-0.12 to -0.04)	110376(70345 to 160712)	19.44(12.31 to 28.32)	264211(168167 to 385966)	18.88(12.00 to 27.60)	-0.09(-0.13 to -0.05)
Sex															
Female	509415(289592 to 808612)	148.51(84.34 to 235.90)	1223164(703219 to 1928567)	154.74(88.99 to 243.99)	0.14(0.09 to 0.20)	688771(535761 to 889372)	213.12(165.56 to 276.26)	1631172(1273752 to 2097317)	205.03(160.08 to 263.58)	-0.12(-0.15 to -0.09)	67568(42559 to 99540)	20.75(13.03 to 30.54)	158398(99814 to 233806)	19.92(12.56 to 29.42)	-0.12(-0.16 to -0.09)
Male	286251(171794 to 443750)	100.72(60.39 to 155.57)	793512(486031 to 1201117)	121.44(74.42 to 183.43)	0.62(0.58 to 0.66)	428292(355666 to 515943)	167.88(138.52 to 204.15)	1067093(872966 to 1303244)	174.07(141.87 to 213.71)	0.13(0.08 to 0.18)	42808(27954 to 60692)	16.55(10.75 to 23.49)	105813(68744 to 151716)	17.12(11.09 to 24.56)	0.12(0.07 to 0.17)
Socio-demographic index															
High SDI	405559(240033 to 622587)	213.57(126.21 to 328.74)	940457(567473 to 1430884)	251.12(151.80 to 382.38)	0.79(0.76 to 0.83)	683427(539612 to 864981)	363.52(287.38 to 460.33)	1573623(1233308 to 2000395)	396.38(311.78 to 501.41)	0.30(0.26 to 0.35)	67258(42485 to 98059)	35.72(22.56 to 52.01)	152985(96771 to 224153)	38.83(24.62 to 56.88)	0.29(0.25 to 0.33)
High-middle SDI	184212(106217 to 293153)	115.75(66.44 to 184.50)	391324(232101 to 609808)	115.78(68.66 to 180.43)	0.34(0.23 to 0.45)	255851(207122 to 316995)	174.79(140.47 to 218.94)	512186(415075 to 634659)	154.66(125.12 to 192.10)	-0.33(-0.44 to -0.21)	25376(16315 to 36602)	17.13(10.95 to 24.76)	50507(32487 to 72804)	15.21(9.76 to 21.92)	-0.31(-0.44 to -0.19)
Middle SDI	105376(57864 to 174382)	66.92(36.78 to 110.52)	412589(235158 to 664543)	94.88(54.12 to 152.68)	1.31(1.21 to 1.40)	94888(76032 to 118985)	68.59(54.77 to 86.16)	377917(301144 to 476324)	93.14(74.00 to 117.75)	1.06(0.97 to 1.16)	9519(6111 to 13865)	6.77(4.33 to 9.83)	37557(23942 to 54798)	9.17(5.82 to 13.37)	1.07(0.97 to 1.18)
Low-middle SDI	80078(43539 to 133386)	86.60(47.05 to 144.21)	215968(122125 to 349414)	97.11(54.99 to 156.99)	0.32(0.30 to 0.34)	65968(51434 to 84901)	81.70(63.53 to 105.46)	187879(147131 to 239225)	93.45(73.08 to 119.29)	0.32(0.27 to 0.36)	6542(4138 to 9613)	7.97(5.04 to 11.70)	18526(11831 to 27287)	9.10(5.80 to 13.39)	0.32(0.28 to 0.37)
Low SDI	19383(10861 to 31549)	57.97(32.49 to 94.20)	54638(31654 to 87435)	74.00(42.88 to 118.39)	0.65(0.63 to 0.67)	15736(12426 to 20059)	56.12(44.14 to 71.70)	44624(35096 to 56821)	69.54(54.62 to 88.54)	0.56(0.50 to 0.62)	1564(990 to 2321)	5.46(3.46 to 8.06)	4435(2809 to 6570)	6.80(4.32 to 10.03)	0.57(0.51 to 0.64)
Region															
Andean Latin America	972(594 to 1497)	29.44(17.98 to 45.36)	4138(2545 to 6307)	42.29(26.01 to 64.47)	1.17(1.14 to 1.20)	1037(856 to 1263)	33.04(27.28 to 40.26)	4415(3624 to 5398)	45.88(37.64 to 56.13)	1.05(1.00 to 1.10)	105(65 to 157)	3.32(2.06 to 4.98)	443(276 to 662)	4.59(2.86 to 6.86)	1.04(0.91 to 1.17)
Australasia	9482(5729 to 14342)	240.75(144.98 to 365.34)	32917(20202 to 48957)	330.49(203.13 to 491.30)	1.03(0.98 to 1.09)	15289(11964 to 19650)	399.09(312.09 to 514.10)	51460(39951 to 66957)	501.96(390.92 to 650.53)	0.74(0.67 to 0.80)	1500(942 to 2194)	39.00(24.45 to 57.04)	4992(3115 to 7384)	48.98(30.57 to 72.44)	0.73(0.66 to 0.80)
Caribbean	2805(1712 to 4293)	67.57(41.21 to 103.44)	9196(5674 to 14055)	99.75(61.61 to 152.35)	1.26(1.22 to 1.30)	2506(1982 to 3184)	63.96(50.54 to 81.30)	8854(7013 to 11238)	94.09(74.49 to 119.46)	1.25(1.20 to 1.30)	252(155 to 383)	6.39(3.94 to 9.67)	872(539 to 1286)	9.29(5.74 to 13.71)	1.21(1.15 to 1.26)
Central Asia	2224(1360 to 3395)	27.53(16.85 to 42.06)	4329(2674 to 6586)	31.38(19.41 to 47.71)	0.45(0.37 to 0.53)	2969(2512 to 3498)	39.01(32.99 to 46.03)	5164(4338 to 6155)	40.65(34.09 to 48.60)	0.16(0.06 to 0.27)	300(193 to 432)	3.93(2.52 to 5.66)	519(334 to 749)	4.05(2.61 to 5.87)	0.13(0.03 to 0.23)
Central Europe	42549(25874 to 64921)	170.31(103.42 to 260.32)	54402(33407 to 83687)	140.37(86.12 to 216.24)	-0.60(-0.69 to -0.51)	41560(33233 to 51826)	179.36(142.83 to 224.86)	57013(46013 to 70724)	143.38(115.88 to 177.67)	-0.71(-0.80 to -0.61)	4112(2634 to 5976)	17.56(11.22 to 25.48)	5628(3617 to 8124)	14.19(9.12 to 20.48)	-0.67(-0.77 to -0.57)
Central Latin America	11188(6425 to 17983)	85.79(49.34 to 137.67)	26583(15657 to 41853)	64.09(37.79 to 100.87)	-0.94(-1.01 to -0.88)	10882(8875 to 13427)	89.23(72.70 to 110.19)	26744(21818 to 32904)	65.91(53.71 to 81.17)	-0.98(-1.04 to -0.91)	1084(699 to 1561)	8.82(5.68 to 12.69)	2657(1704 to 3871)	6.53(4.19 to 9.52)	-0.97(-1.04 to -0.90)
Central Sub-Saharan Africa	1080(649 to 1688)	32.50(19.49 to 50.89)	3075(1872 to 4727)	40.09(24.38 to 61.96)	0.66(0.61 to 0.71)	829(657 to 1055)	31.22(24.82 to 39.67)	2389(1891 to 3059)	37.95(29.97 to 48.62)	0.62(0.55 to 0.69)	83(50 to 129)	3.06(1.85 to 4.69)	241(143 to 371)	3.75(2.26 to 5.72)	0.64(0.54 to 0.75)
East Asia	84343(45037 to 141138)	62.66(33.39 to 105.00)	361287(202732 to 587471)	100.56(56.50 to 163.58)	1.62(1.38 to 1.87)	76860(61589 to 96444)	66.14(52.81 to 83.24)	338408(270032 to 425371)	100.19(79.63 to 126.50)	1.43(1.17 to 1.70)	7802(4990 to 11406)	6.59(4.20 to 9.64)	33802(21486 to 49425)	9.91(6.28 to 14.47)	1.40(1.14 to 1.67)
Eastern Europe	33105(18448 to 54429)	67.25(37.38 to 110.58)	46961(27148 to 75047)	76.14(44.06 to 121.88)	0.42(0.24 to 0.59)	43949(36855 to 52546)	93.16(78.08 to 111.54)	61104(51209 to 72830)	98.42(82.46 to 117.34)	0.19(0.07 to 0.31)	4380(2873 to 6252)	9.25(6.06 to 13.21)	6062(3987 to 8612)	9.76(6.42 to 13.88)	0.19(0.07 to 0.31)
Eastern Sub-Saharan Africa	3725(2166 to 5936)	33.76(19.67 to 53.71)	9708(5774 to 15159)	40.96(24.37 to 64.05)	0.63(0.61 to 0.64)	3015(2384 to 3853)	32.53(25.69 to 41.64)	7881(6230 to 10033)	38.67(30.52 to 49.27)	0.55(0.53 to 0.57)	303(191 to 454)	3.20(2.03 to 4.78)	790(497 to 1176)	3.81(2.40 to 5.65)	0.56(0.53 to 0.58)
High-income Asia Pacific	49660(27991 to 80092)	146.68(82.35 to 237.25)	125294(74244 to 193737)	154.00(92.10 to 237.09)	0.16(0.12 to 0.19)	91357(73307 to 112882)	280.39(224.15 to 348.27)	236746(188166 to 297825)	264.01(212.14 to 328.14)	-0.20(-0.24 to -0.16)	9133(5869 to 13296)	27.86(17.85 to 40.56)	23280(14691 to 34042)	26.33(16.78 to 38.52)	-0.19(-0.24 to -0.14)
High-income North America	115496(64186 to 187123)	191.32(106.31 to 310.67)	358768(208946 to 561090)	311.12(181.40 to 486.97)	1.61(1.54 to 1.68)	204945(159149 to 262810)	332.44(258.69 to 425.78)	597292(461720 to 765565)	498.08(385.03 to 638.09)	1.32(1.24 to 1.40)	20082(12664 to 29405)	32.63(20.61 to 47.74)	57660(36364 to 84767)	48.25(30.43 to 71.02)	1.28(1.20 to 1.36)
North Africa and Middle East	7829(4605 to 12466)	30.71(18.02 to 48.85)	33273(19895 to 51643)	49.61(29.65 to 77.04)	1.56(1.50 to 1.61)	8335(6841 to 10157)	35.81(29.26 to 43.86)	33173(27201 to 40699)	53.06(43.26 to 65.45)	1.27(1.24 to 1.31)	836(542 to 1211)	3.54(2.29 to 5.13)	3298(2123 to 4787)	5.22(3.34 to 7.58)	1.25(1.17 to 1.32)
Oceania	177(101 to 291)	47.03(26.66 to 77.17)	650(377 to 1046)	67.51(39.18 to 108.61)	1.17(1.12 to 1.21)	147(117 to 185)	46.76(36.82 to 59.30)	545(432 to 689)	65.87(51.61 to 84.09)	1.11(1.05 to 1.16)	15(9 to 23)	4.58(2.73 to 6.95)	54(33 to 82)	6.44(3.89 to 9.64)	1.10(1.02 to 1.17)
South Asia	104013(55418 to 175131)	123.06(65.53 to 207.05)	325781(181910 to 531698)	142.32(79.56 to 232.28)	0.47(0.45 to 0.48)	81934(62627 to 107418)	113.95(86.82 to 149.93)	272853(209478 to 353974)	133.87(102.64 to 174.24)	0.52(0.50 to 0.54)	8099(5074 to 11983)	11.04(6.91 to 16.31)	26817(16942 to 39964)	12.97(8.17 to 19.27)	0.52(0.50 to 0.54)
Southeast Asia	21454(12203 to 34666)	56.17(31.96 to 90.67)	73536(43431 to 115614)	71.93(42.49 to 113.15)	0.81(0.77 to 0.86)	18737(14950 to 23640)	55.96(44.64 to 70.64)	63675(51020 to 79740)	69.21(55.33 to 86.84)	0.70(0.65 to 0.75)	1880(1192 to 2778)	5.53(3.50 to 8.12)	6373(4046 to 9361)	6.84(4.33 to 10.03)	0.70(0.64 to 0.75)
Southern Latin America	8289(5090 to 12442)	108.47(66.47 to 163.06)	18219(11261 to 27231)	120.60(74.58 to 180.23)	0.34(0.31 to 0.37)	14592(11958 to 17885)	197.71(161.35 to 243.49)	33001(26792 to 40750)	216.02(175.59 to 266.39)	0.29(0.24 to 0.33)	1454(926 to 2135)	19.60(12.45 to 28.78)	3249(2050 to 4748)	21.30(13.45 to 31.12)	0.27(0.21 to 0.33)
Southern Sub-Saharan Africa	855(482 to 1392)	20.50(11.55 to 33.38)	1783(1036 to 2842)	19.78(11.48 to 31.55)	-0.15(-0.23 to -0.06)	885(711 to 1106)	22.63(18.15 to 28.37)	1713(1382 to 2141)	20.78(16.73 to 26.05)	-0.28(-0.32 to -0.25)	89(56 to 130)	2.24(1.42 to 3.29)	170(108 to 251)	2.04(1.30 to 3.01)	-0.32(-0.38 to -0.26)
Tropical Latin America	11225(6041 to 18853)	78.11(42.00 to 131.28)	36857(20874 to 59834)	85.23(48.28 to 138.33)	0.29(0.27 to 0.32)	12245(10048 to 14945)	91.45(74.76 to 112.12)	39205(31956 to 48467)	92.45(75.28 to 114.38)	0.04(-0.01 to 0.08)	1217(788 to 1763)	9.00(5.81 to 13.04)	3871(2465 to 5657)	9.11(5.80 to 13.30)	0.04(0.02 to 0.06)
Western Europe	279967(168852 to 422938)	273.99(165.11 to 415.26)	476120(292510 to 714381)	273.50(168.46 to 410.00)	0.00(-0.02 to 0.03)	480635(377746 to 612403)	476.30(375.88 to 605.82)	845452(663963 to 1076434)	461.28(365.17 to 581.58)	-0.11(-0.13 to -0.08)	47214(29658 to 68755)	46.74(29.42 to 67.93)	82309(51728 to 120843)	45.37(28.63 to 66.53)	-0.10(-0.14 to -0.06)
Western Sub-Saharan Africa	5230(3012 to 8402)	39.55(22.75 to 63.52)	13798(8141 to 21735)	49.20(29.06 to 77.53)	0.70(0.68 to 0.73)	4356(3413 to 5593)	37.89(29.67 to 48.72)	11181(8839 to 14201)	46.03(36.39 to 58.47)	0.63(0.60 to 0.66)	435(276 to 644)	3.73(2.37 to 5.49)	1123(712 to 1668)	4.56(2.89 to 6.74)	0.64(0.62 to 0.67)

*Notes*: Rates are reported per 100,000 person-years. Data in parentheses are 95% uncertainty intervals for cases and age-standardized rates of incidence, prevalence and YLDs, and 95% confidence intervals for AAPCs. Abbreviations: YLDs, years lived with disability; AAPC, average annual percent change; SDI, socio-demographic index; UI, uncertainty interval; ASYR, age-standardized years lived with disability rate; ASPR, age-standardized prevalence rate; ASIR, age-standardized incidence rate.

### 3.3 Burden trends by sex

**Fig 2:**
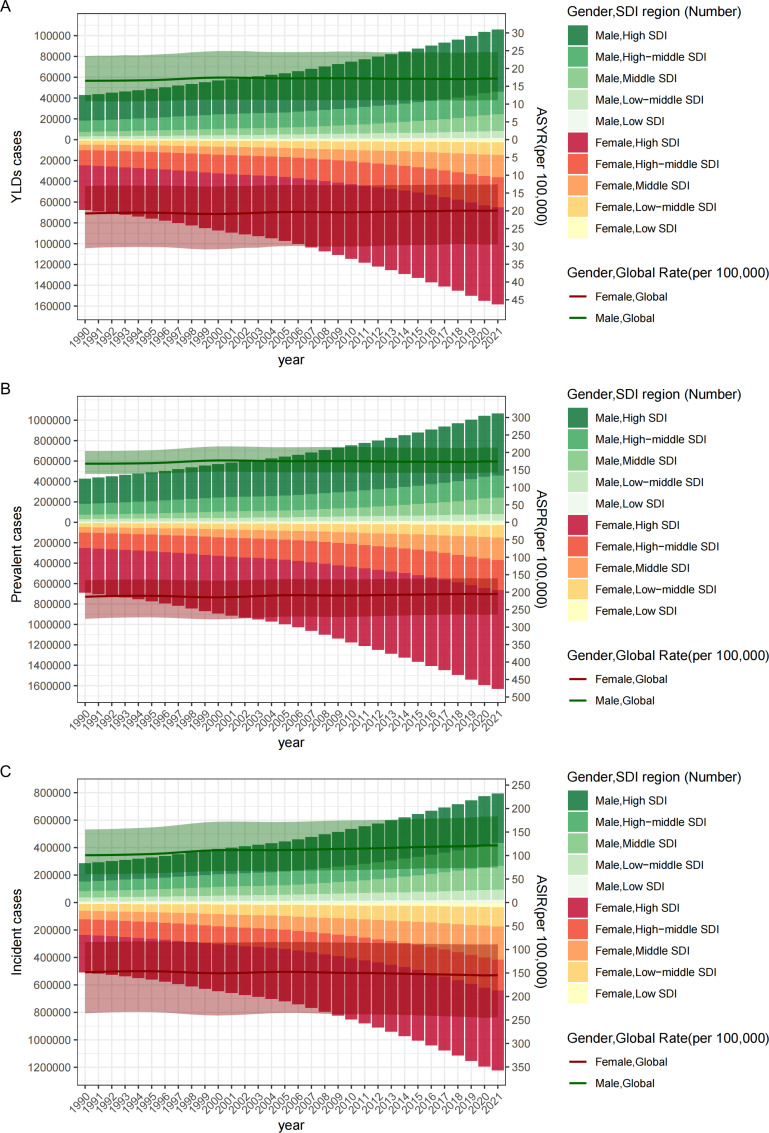
Correlations between male and female vertebral fracture individuals aged 55 and above and SDI regional from 1990 to 2021. *Notes*: ASYR and number of YLD of vertebral fractures due to falls among males and females aged 55 and older at the global and SDI regions from 1990 to 2021 (A). ASPR and number of prevalent cases of vertebral fractures due to falls among males and females aged 55 and older at the global and SDI regions from 1990 to 2021 (B). ASIR and number of incident cases of vertebral fractures due to falls among males and females aged 55 and older at the global and SDI regions from 1990 to 2021 (C). Abbreviations: YLDs, years lived with disability; ASYR, age-standardized years lived with disability rate; ASPR, age-standardized prevalence rate; ASIR, age-standardized incidence rate; SDI, socio-demographic index.

**Fig 3:**
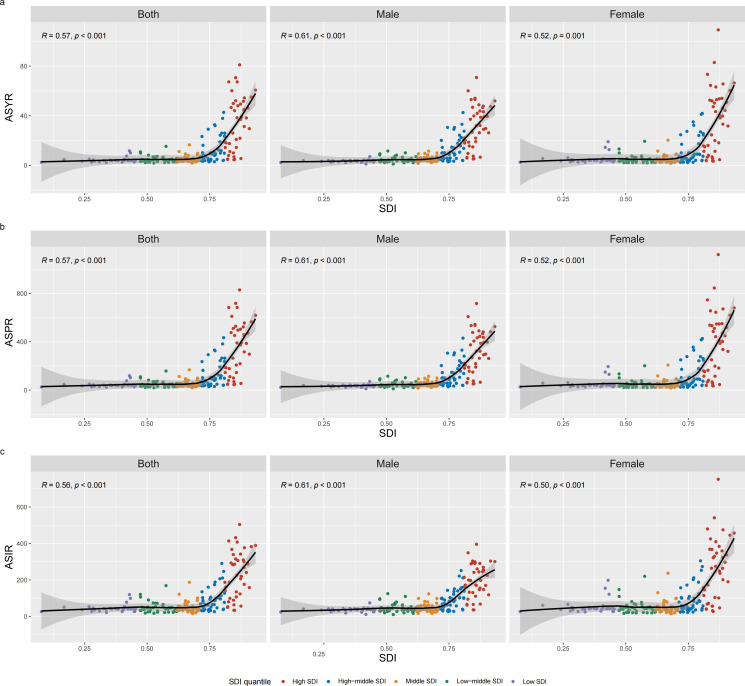
Correlations between ASIR, ASPR, and ASYR vertebral fracture individuals aged 55 and above and SDI at the national level. *Notes*: In 2021, ASYR of vertebral fractures among individuals aged 55 and above in 204 countries, by SDI, the total population (a), males (b), and females (c). In 2021, ASPR of vertebral fractures among individuals aged 55 and above in 204 countries, by SDI, the total population (d), males (e), and females (f). In 2021, ASIR of vertebral fractures among individuals aged 55 and above in 204 countries, by SDI, the total population (g), males (h), and females (i). Abbreviations: YLDs, years lived with disability; ASYR, age-standardized years lived with disability rate; ASPR, age-standardized prevalence rate; ASIR, age-standardized incidence rate; SDI, socio-demographic index.

Gender analysis shows that in 2021, the number of new cases of vertebral fractures in females is 1.22 million, while in males, it is 793,512. There were approximately 1.63 million prevalent cases of vertebral fractures among women aged 55 and above worldwide, and approximately 1.07 million prevalent cases among men. Both populations have shown significant growth compared to 1990. In 2021, women accounted for 158,398 YLDs more than men accounted for 105,813 YLDs. The ASIR of female vertebral fractures is 154.74 cases per 100,000 people (95% UI 88.99-243.99), higher than that of male fractures at 121.44 cases per 100,000 people (95% UI: 74.42-183.43). It is evident that the ASIR of vertebral fractures caused by falls in both men and women is on the rise. In 2021, female ASPR was 205.03 per 100,000 people (95% UI: 160.08–263.58), higher than male ASPR of 174.07 (95% UI: 141.87–213.71). But compared to 1990, female ASPR showed a downward trend (AAPC: -0.12; 95% CI: -0.15 to -0.09), while male ASPR showed an upwardly trend (AAPC: 0.13; 95% CI: 0.08 to 0.18). In 2021, the male ASYR was 17.12 (95% UI: 11.09–24.56) per 100,000 people, and the female ASYR was 19.92 (95% UI: 12.56-29.42). Similarly, compared with 1990, male ASYR showed an upward trend (AAPC: 0.12; 95% CI: 0.07 to 0.17), while female ASYR showed a downward trend (AAPC: -0.12; 95% CI: -0.16 to -0.09) (Table 1). The inflection points of ASYR, ASPR, and ASIR, categorized by gender, can be found in Fig 1 (Males: Fig 1(a), Fig 1(d), and Fig 1(g); Females: Fig 1(b), Fig 1(e), and Fig 1(h)). The numbers of YLDs, prevalent cases, and incident cases of vertebral fractures, categorized by gender, at the global level and across SDI regions over the years, are represented using a bar chart (Fig 2). The gender differences in ASYR, ASPR, and ASIR in relation to SDI at the national level are shown in the figure for 2021 (Males: Fig 3(b), Fig 3(e), and Fig 3(h); Females: Fig 3(c), Fig 3(f), and Fig 3(i)).

### 3.4 Burden trends by region and country

**Fig 4:**
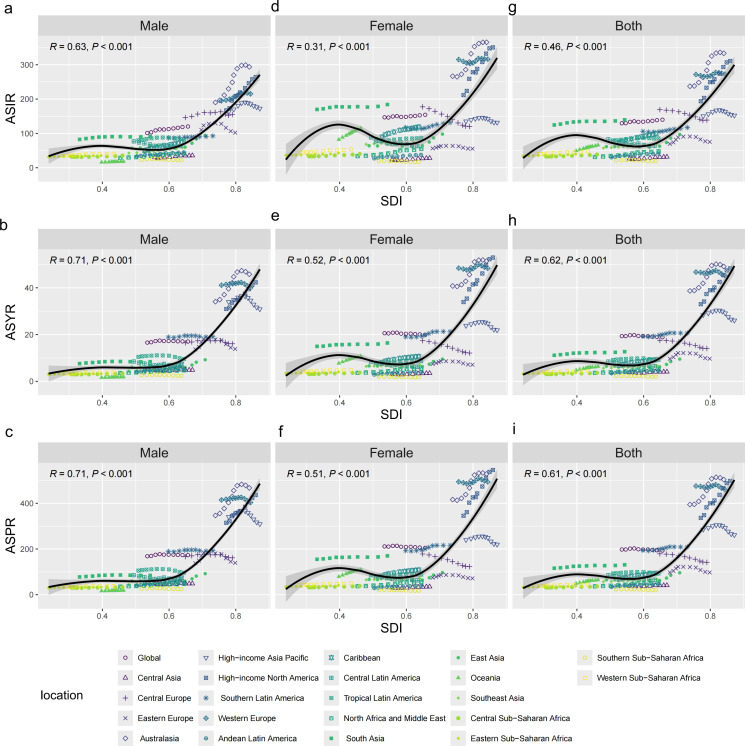
Correlations between ASIR, ASPR, and ASYR of global vertebral fractures due to falls among males and females aged 55 and older and SDI at the regional level. *Notes*: ASIR of vertebral fractures due to falls among males aged 55 and older at the global level and 21 regions, by SDI, from 1990 to 2021 (a). ASIR of vertebral fractures due to falls among females aged 55 and older at the global level and 21 regions, by SDI, from 1990 to 2021 (d). ASIR of vertebral fractures due to falls among the total population aged 55 and older at the global level and 21 regions, by SDI, from 1990 to 2021 (g). ASYR of vertebral fractures due to falls among males aged 55 and older at the global level and 21 regions, by SDI, from 1990 to 2021 (b). ASYR of vertebral fractures due to falls among females aged 55 and older at the global level and 21 regions, by SDI, from 1990 to 2021 (e). ASYR of vertebral fractures due to falls among the total population aged 55 and older at the global level and 21 regions, by SDI, from 1990 to 2021 (h). ASPR of vertebral fractures due to falls among males aged 55 and older at the global level and 21 regions, by SDI, from 1990 to 2021 (c). ASPR of vertebral fractures due to falls among females aged 55 and older at the global level and 21 regions, by SDI, from 1990 to 2021 (f). ASPR of vertebral fractures due to falls among the total population aged 55 and older at the global level and 21 regions, by SDI, from 1990 to 2021 (i). Abbreviations: YLDs, years lived with disability; ASYR, age-standardized years lived with disability rate; ASPR, age-standardized prevalence rate; ASIR, age-standardized incidence rate; SDI, socio-demographic index.

At the regional level, East Asia, high-income North America, and North Africa and Middle East exhibited the most significant increases in ASIR from 1990 to 2021 (Fig 4(g) and Table 1), with the corresponding AAPCs both appearing to be greater than 1.5. Similarly, ASPR and ASYR have the fastest AAPC growth in these three regions (Fig 4(i), (h) and Table 1). In 2021, the regions with the highest number of incident cases of vertebral fractures among elderly people aged 55 and above were Western Europe, East Asia, and high-income North America. The three regions with the highest number of prevalent cases and YLDs were Western Europe, High-income North America, and East Asia. In 1990, Western Europe, High-income North America, and South Asia were the top three regions the with highest new cases. Western Europe, High-income North America, and High-income Asia Pacific were the top three regions with the highest prevalent cases and YLDs numbers. Regarding the regional differences in ASIR, ASPR, and ASYR, the top three regions in 2021 are Australasia, High incoming North America, and Western Europe. In 1990, Western Europe, Australasia, and High-income North America had the top three ASIR, ASPR, and ASYR (Table 1). The gender differences in ASIR, ASYR, and ASPR in relation to SDI at the regional level are shown in the figure (Males: Fig 4(a), Fig 4(b), and Fig 4(c); females: Fig 4(d), Fig 4(e), and Fig 4(f)).

The three countries with the highest new cases in 2021 are China, the United States of America, and India. This trend is particularly pronounced in these three countries due to their large population sizes, which have a considerable impact on the aggregated data and overall burden estimates. The highest number of prevalent cases and YLDs are in the United States, China, and India. In 1990, United States of America, India, and China were the top three countries the with highest new cases. United States of America, Germany, and Italy were the top three countries with the highest prevalent cases and YLDs number. In 2021, the three countries with the highest ASIR and ASPR are Andorra, Belgium and Greenland. The three countries with the highest ASYR are Andorra, Belgium, and Finland. In 1990, Switzerland, Greenland, and Andorra had the top three ASIR, ASPR, and ASYR (Fig 3 and S1 Table).

### 3.5 Burden trends association with the SDI

With regard to SDI regions, the ASIR, ASPR, and ASYR among low SDI, low-middle SDI, middle SDI, and high SDI regions all exhibited increasing trends from 1990 to 2021. In 2021, the ASIR, ASPR, and ASYR are highest in the high SDI region (high SDI ASIR = 251.12 [95% UI: 151.80 to 382.38]; ASPR = 396.38 [95% UI: 311.78 to 501.41]; ASYR = 38.83 [95% UI: 24.62 to 56.88]) and lowest in the low SDI region (low SDI ASIR = 74.00 [95% UI: 42.88 to 118.39]; ASPR = 69.54 [95% UI: 54.62 to 88.54]; ASYR = 6.80 [95% UI: 4.32 to 10.03]). In addition, the high SDI region had the highest number of new cases, prevalent cases, and YLDs in 1990 and 2021, indicating it had the highest burden (Fig 4).

A significant correlation between SDI and ASIR, ASPR, and ASYR in the burden of vertebral fractures caused by falls. ASIR, ASPR, and ASYR increase with the increase of SDI when the SDI level is high, and the upward trend accelerates significantly in high SDI areas. At the regional level, the observed Australasia, Western Europe, and high-income North America ASIR, ASPR, and ASYR slightly exceeded the fitted curve. The observed values of ASIR, ASPR, and ASYR in Andorra, Belgium, Greenland, and Finland slightly exceeded the fitted curve.

### 3.6 Predictions of vertebral fractures burden among individuals aged 55 and older to 2035

**Fig 5:**
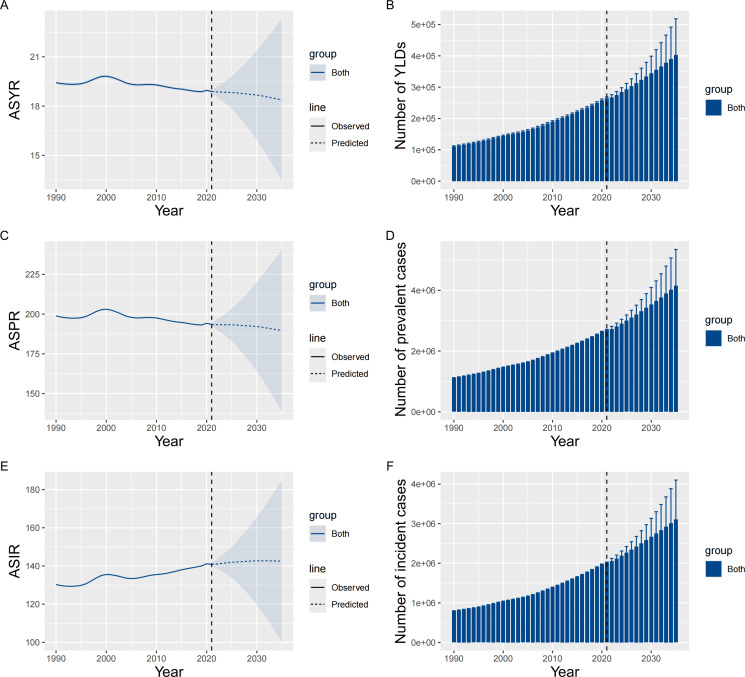
Projected trends in vertebral fractures due to falls among individuals aged 55 and older: ASYR (A), ASPR (C), ASIR (E), and the number of YLDs (B), prevalent cases (D), and incident cases (F) to 2035 based on BAPC models. Abbreviations: YLDs, years lived with disability; ASYR, age-standardized years lived with disability rate; ASPR, age-standardized prevalence rate; ASIR, age-standardized incidence rate.

From 2022 to 2035, the number of cases and YLDs will continue to increase worldwide. At the same time, ASPR and ASYR of vertebral fractures caused by falls among individuals aged 55 and older follow a downward trend. ASIR follow an upward trend. The ASPR and ASYR in the overall population would decrease from 193.50, 18.88 per 100,000 in 2021 to 189.62, 18.37 per 100,000 in 2035, respectively. ASIR increase from.140.77 per 100,000 in 2021 to 142.47 per 100,000 in 2035 (Fig 5) (Table 1 and S2 Table). The number of incident cases, prevalent cases, and YLDs in men would increase from 793,512, 1,067,093, 105,813 in 2021 to 1,201,206, 1,612,141, 158,981 in 2035, respectively. Women would increase from 1,223,164, 1,631,172, 158,398 in 2021 to 1,909,513, 2,566,720, 245,957 in 2035, respectively. The ASPR and ASYR in men would decrease from 174.07, 17.12 per 100,000 in 2021 to 172.55, 16.95 per 100,000 in 2035, respectively; ASIR increase from 121.44 per 100,000 in 2021 to 124.49 per 100,000 in 2035. The ASPR and ASYR in women would decrease from 205.03, 19.92 per 100,000 in 2021 to 201.63, 19.39 per 100,000 in 2035, respectively; ASIR increase from 154.74 per 100,000 in 2021 to 155.84 per 100,000 in 2035 (Table 1 and S1 Fig).

## 4 Discussion

Previous GBD studies mainly focused on the global burden of vertebral fractures in the general population, neglecting specific risk factors and high-risk populations. This study is the first to study the temporal trends in incidence, prevalence, and YLDs of vertebral fractures due to falls among individuals aged 55 and older globally from 1990 to 2021, including comparisons by gender, region, and country. The global burden of vertebral fractures attributable to falls in individuals aged 55 and above has demonstrated an upward trend, exhibiting notable gender and regional variability. Socioeconomic factors play a crucial role, with the highest burden observed in high SDI regions, Western Europe, and the United States. In high-SDI regions, the burden shows a positive correlation with SDI. Additionally, the burden remains higher among females, with a greater number of female cases compared to males. While ASPR and ASYR have generally risen in males, they have decreased in females. Predictive analysis indicated that the global burden of vertebral fractures caused by falls among individuals aged 55 and older was projected to remain heavy in the future.

With the extension of life expectancy and the decline in birth rates, the global elderly population is steadily increasing. This demographic shift has also driven the rising prevalence of osteoporosis. Osteoporosis is a systemic skeletal disorder marked by reduced bone mass and compromised microarchitecture, leading to heightened bone fragility and fracture risk. Osteoporosis is defined based on bone mineral density (BMD) measurements [3]. While postmenopausal osteoporosis is still common, the impact of secondary osteoporosis on the population cannot be ignored [26]. A U.S. study reported that the prevalence of osteoporosis among people aged 50 and over reached 12.6% between 2017 and 2018. Fractures caused by osteoporosis posed a vast economic and medical burden, with the United States reporting approximately 2 million cases annually and an estimated cost of $17 billion [3]. However, adherence to pharmacological treatment for osteoporosis remains low [27]. Vertebral compression fractures are a common complication of osteoporosis [28]. Among elderly individuals, falls are the predominant risk factor for vertebral fractures [12]. One-third of community-dwelling adults aged ≥ 65 years and half of those aged > 80 years experience at least one fall annually, and 10% of elderly individuals fall at least twice yearly [29]. Osteoporotic individuals, due to biochemical changes, may experience postural instability and decreased activity levels, increasing their risk of falls [30]. Falls typically result from a combination of intrinsic factors, such as balance impairments and vision issues, and extrinsic factors, like tripping or slipping [29]. Controlled trials have demonstrated that training in balance and gait patterns can reduce the rate and number of falls [31]. Performing at least 3 hours of balance and functional training per week has reduced the fall rate by 42% in adults over 65 years old [32]. Interventions addressing fall-related risk factors can help reduce fall incidence. By considering osteoporosis risk factors and fall tendencies, the risk of fractures can be qualitatively assessed [33]. Meanwhile, timely and regular pharmacological or non-pharmacological anti-osteoporosis treatments are of great importance in reducing vertebral fractures in patients with osteoporosis [34]. Education for both patients and physicians may further improve the diagnosis and treatment of osteoporosis, thereby reducing the risk of fractures [35, 36]. This can assist in developing preventive policies for high-risk populations.

In 2021, the number of cases and YLDs due to vertebral fractures caused by falls in individuals aged 55 and above increased compared to 1990. This increase is primarily attributed to population aging. Although ASYR and ASPR have shown a decreasing trend overall, ASIR has continued to rise, especially since 2005. This trend warrants the attention of researchers and relevant departments. Therefore, it is essential to implement appropriate prevention programs globally, especially in regions with limited healthcare resources and in low- and middle-income countries (LMICs), to ensure that high-risk elderly populations have adequate access to healthcare services to address these injuries.

Gender analysis revealed that, compared to 1990, both the new cases and prevalent cases of vertebral fractures due to falls increased for both males and females, with females having higher case numbers than males. The greater prevalence of osteoporosis in females likely accounts for this disparity [3]. Low muscle mass increases the risk of falls [37], which may contribute to the higher fall risk in females. Moreover, the female spine is more vulnerable to injury under equivalent external forces. On the other hand, age-standardized indicators for females were higher than those for males. ASIR exhibited an upward trend in both genders, while ASPR and ASYR generally decreased in females but increased in males. A study on fall-related injuries revealed that men sustained more injuries than women, with 29% of injuries involving the spine [38]. An epidemiological study on workplace injuries demonstrated a higher fall rate among men than women. Compared to women, men are underdiagnosed and undertreated for osteoporosis [39, 40], have poorer adherence to osteoporosis medications [41, 42], and are more likely to consume excessive alcohol [43], further heightening their risk of vertebral fractures. Therefore, while continuing to strengthen the management of osteoporosis in women, future prevention efforts should shift focus toward men, prioritizing increased awareness, diagnosis, and treatment of male osteoporosis, as well as targeting fall-related risk factors.

Socioeconomic status has a momentous impact on the burden of vertebral fractures due to falls in individuals aged 55 and above. At the regional level, high SDI regions exhibited the highest numbers of cases and YLDs, as well as the highest ASR values in both 1990 and 2021. In contrast, these indicators were lowest in the low SDI regions. The burden is higher in Western Europe, high-income North America, and East Asia. This disparity may result from longer life expectancy, higher levels of physical activity, and larger population base, in high SDI regions. In contrast, low SDI regions exhibit the opposite trend. Differences in universal healthcare coverage and access to medical services between high and low SDI regions may also play a role. Furthermore, the diagnosis rate of vertebral fractures and the positive rate among asymptomatic patients may be higher in high SDI regions [44]. For instance, the limited number of dual-energy x-ray absorptiometry (DXA) in Africa contributes to lower detection rates. Lower life expectancy and insufficient healthcare services further diminish motivation for fracture risk assessment and treatment [45]. The lack of functioning survey institutions and relevant services in Africa also contributes to data inaccuracies [46]. Urbanization is greater in high SDI regions, and studies suggest that older adults in urban areas face higher fall rates and a greater fear of falling compared to rural counterparts [47]. However, for the ASYR results, a high detection rate does not necessarily translate to reduced adverse outcomes. This could be related to side effects and adherence to medication [48], and the financial burden [10]. This underscores the need for greater attention to disabilities caused by fall-induced vertebral fractures.

At the national level, the burden of disease remains high in the United States, China, and India. In the U.S., over 14 million adults aged 65 and older report falls annually [49], with vertebral fracture risks from falls being significant. Additionally, U.S. resident physicians lack sufficient awareness of osteoporosis, resulting in delayed treatment for patients [50]. A cross-sectional study of Chinese adults revealed a high prevalence of osteoporosis and vertebral fractures [51]. Several studies indicate that the prevalence of osteoporosis among Indian women ranges from 8% to 62%. Inadequate dietary calcium intake, low levels of vitamin D, and lifestyle changes contribute to the development of osteoporosis [52], which may exacerbate the disease burden in these countries.

It is worth noting that in high-income countries, the use of private vehicles as a primary means of transportation may contribute to a higher incidence of vertebral fractures caused by traffic accidents. Although the present study focuses on falls as the primary cause of vertebral fractures, the role of traffic accidents should not be overlooked, as it represents a significant risk factor for vertebral injuries in these regions. This highlights the need for further research into the impact of transportation-related injuries on the burden of vertebral fractures, particularly in high-income settings. Studies from Italy, the Netherlands, and Germany indicate that traffic accidents remain a significant factor in causing vertebral fractures [53–55].

COVID-19 may directly and indirectly impact osteoclasts and osteoblasts, contributing to osteoporosis through factors such as vitamin D deficiency, pro-inflammatory cytokines, and glucocorticoid use [56]. A Hong Kong-based study confirmed this, showing an increased risk of severe osteoporotic fractures in the acute and post-acute phases of COVID-19 infection among elderly individuals, partly due to an elevated risk of falls [57], aligning with our findings.

However, it must be acknowledged that this study has certain limitations. First, while the GBD 2021 data is considered high-quality, in regions severely affected by the COVID-19 pandemic and in many LMICs, the lack of original data and statistical difficulties may have introduced potential errors. Additionally, GBD methods rely on modeled data, adding uncertainty to population estimates. Second, the study focused primarily on patients with fractures seeking medical care, potentially excluding asymptomatic or mildly symptomatic cases. Third, manual calculation of the ASR for vertebral fractures due to falls in individuals aged 55 and older mitigated the impact of age structure differences but influenced the derivation of uncertainty intervals, affecting data quality and availability. Finally, although this study provides a global perspective related to the burden of vertebral fractures caused by falls in older adults, differences between populous countries and small populations may distort conclusions and may not reflect the realities of specific regions.

## 5 Conclusions

In conclusion, from 1990 to 2021, the global burden of vertebral fractures caused by falls in individuals aged 55 and above has increased. The numbers of incident cases, prevalent cases, and YLDs has increased. The rise in ASIR, in the context of significant population growth, further underscores this trend. Currently, the burden remains heavier for females, but more attention should be directed toward males in the future. The global burden shifts with socioeconomic development, with high SDI regions bearing the greatest impact. Falls remain the main risk factor for vertebral fractures in the elderly. In the future, the world will face increasingly growing healthcare and economic challenges related to this issue. This study provides vital evidence for the public, researchers, and policymakers to raise awareness, prevent falls, reduce the incidence of vertebral fractures in older adults, and promote the implementation of healthy aging strategies.

## Supporting information

S1 Fig Projected trends in vertebral fractures due to falls among males and females aged 55 and older : ASYR (A), ASPR (C), ASIR (E), and the number of YLDs (B), prevalent cases (D), and incident cases (F) to 2035 based on BAPC models. Abbreviations: YLDs, years lived with disability; ASYR, age-standardized years lived with disability rate; ASPR, age-standardized prevalence rate; ASIR, age-standardized incidence rate.
(TIF)

S1 Table Incidence, prevalence and YLDs of vertebral fractures caused by falls among elderly people in 1990 and 2021, and AAPC from 1990 to 2021, by countries. *Notes*: Rates are reported per 100,000 person-years. Data in parentheses are 95% uncertainty intervals for cases and age-standardized rates of incidence, prevalence and YLDs, and 95% confidence intervals for AAPCs. Abbreviations: YLDs, years lived with disability; AAPC, average annual percent change; UI, uncertainty interval; ASYR, age-standardized years lived with disability rate; ASPR, age-standardized prevalence rate; ASIR, age-standardized incidence rate.
(DOCX)

S2 Table Forecast of vertebral fractures caused by falls among elderly people age-standardized incidence, prevalence and YLDs rates and cases globally, to 2035. *Notes*: Rates are reported per 100,000 person-years. Data in parentheses are 95% uncertainty intervals for cases and age-standardized rates of incidence, prevalence and YLDs. ASYR, age-standardized years lived with disability rate; ASPR, age-standardized prevalence rate; ASIR, age-standardized incidence rate; YLDs, years lived with disability; UI, uncertainty interval.
(DOCX)
