# Supplementary figures and images for: The global burden of vertebral fractures caused by falls among individuals aged 55 and older, 1990 to 2021
Source: PLoS One. 2025 Apr 8;20(4):e0318494. doi: 10.1371/journal.pone.0318494 (PMC11978109; doi:10.1371/journal.pone.0318494)

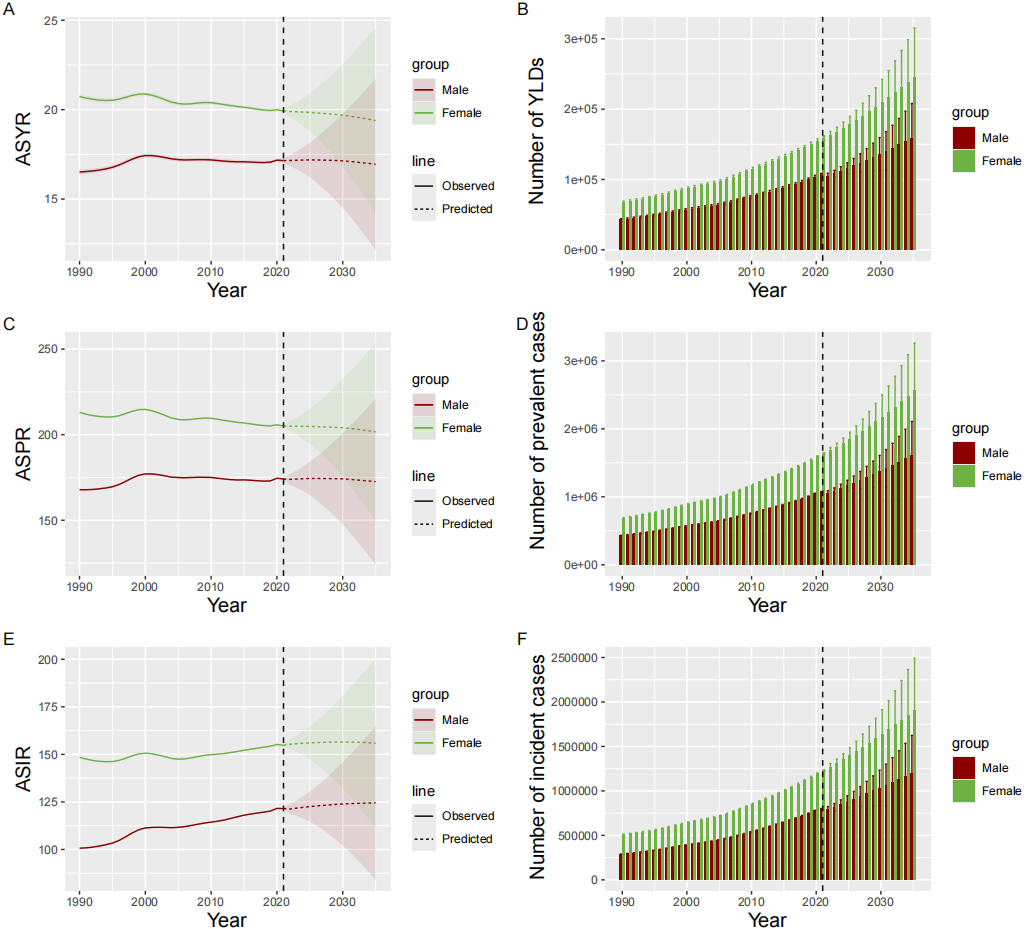

Supplement: S1 Fig — : ASYR (A), ASPR (C), ASIR (E), and the number of YLDs (B), prevalent cases (D), and incident cases (F) to 2035 based on BAPC models. Abbreviations: YLDs, years lived with disability; ASYR, age-standardized years lived with disability rate; ASPR, age-standardized prevalence rate; ASIR, age-standardized incidence rate. (TIF) [file pone.0318494.s001.tif]
