# Supplementary material for: The global burden of vertebral fractures caused by falls among individuals aged 55 and older, 1990 to 2021
Source: PLoS One. 2025 Apr 8;20(4):e0318494. doi: 10.1371/journal.pone.0318494 (PMC11978109; doi:10.1371/journal.pone.0318494)
Supplement: S1 Table — Notes: Rates are reported per 100,000 person-years. Data in parentheses are 95% uncertainty intervals for cases and age-standardized rates of incidence, prevalence and YLDs, and 95% confidence intervals for AAPCs. Abbreviations: YLDs, years lived with disability; AAPC, average annual percent change; UI, uncertainty interval; ASYR, age-standardized years lived with disability rate; ASPR, age-standardized prevalence rate; ASIR, age-standardized incidence rate. (DOCX) [file pone.0318494.s002.docx]

**Supplemental Table 1. Incidence,** **Prevalence and YLDs of vertebral fractures caused by falls among elderly people in 1990 and 2021, and AAPC from 1990 to 2021, by countries.**

| **Location** | **Incidence** | | | | | **Prevalence** | | | | | **YLDs** | | | | |
| --- | --- | --- | --- | --- | --- | --- | --- | --- | --- | --- | --- | --- | --- | --- | --- |
|  | **Incident cases(1990)** | **ASIR(1990)** | **Incident cases(2021)** | **ASIR(2021)** | **AAPC**  **1990–2021** | **Prevalent cases(1990)** | **ASPR(1990)** | **Prevalent cases(2021)** | **ASPR(2021)** | **AAPC**  **1990–2021** | **YLDs(1990)** | **ASYR(1990)** | **YLDs(2021)** | **ASYR( 2021)** | **AAPC**  **1990–2021** |
| **Afghanistan** | 185(107 to 295) | 17.44(10.12 to 27.78) | 237(141 to 373) | 21.21(12.58 to 33.37) | 0.64(0.57 to 0.70) | 208(170 to 253) | 21.26(17.35 to 26.16) | 253(207 to 311) | 24.13(19.62 to 29.76) | 0.41(0.38 to 0.45) | 21(12 to 31) | 2.08(1.22 to 3.14) | 25(15 to 37) | 2.33(1.44 to 3.51) | 0.36(0.28 to 0.43) |
| **Albania** | 113(65 to 184) | 34.66(19.76 to 56.36) | 344(204 to 538) | 44.14(26.21 to 69.13) | 0.80(0.62 to 0.98) | 131(106 to 164) | 42.63(34.43 to 53.63) | 415(340 to 507) | 54.82(44.83 to 67.08) | 0.82(0.67 to 0.98) | 13(8 to 20) | 4.24(2.55 to 6.53) | 42(24 to 64) | 5.48(3.22 to 8.44) | 0.86(0.70 to 1.03) |
| **Algeria** | 562(333 to 886) | 29.43(17.49 to 46.33) | 2107(1269 to 3270) | 38.36(23.14 to 59.58) | 0.86(0.83 to 0.89) | 617(506 to 749) | 35.68(29.02 to 43.99) | 2197(1805 to 2710) | 42.73(34.95 to 52.92) | 0.59(0.56 to 0.62) | 62(36 to 97) | 3.54(2.05 to 5.49) | 220(128 to 344) | 4.22(2.49 to 6.59) | 0.58(0.55 to 0.62) |
| **American Samoa** | 1(1 to 2) | 40.11(24.38 to 62.05) | 4(2 to 6) | 50.93(31.47 to 78.19) | 0.76(0.74 to 0.78) | 1(1 to 1) | 40.78(32.65 to 50.84) | 3(3 to 4) | 50.60(40.49 to 63.28) | 0.68(0.66 to 0.71) | 0(0 to 0) | 4.05(2.34 to 6.52) | 0(0 to 1) | 4.94(2.88 to 7.70) | 0.65(0.60 to 0.69) |
| **Andorra** | 37(22 to 57) | 404.94(240.37 to 628.50) | 139(84 to 210) | 503.90(305.02 to 763.12) | 0.73(0.61 to 0.85) | 59(46 to 75) | 689.01(538.76 to 880.46) | 238(187 to 304) | 829.24(652.38 to 1058.14) | 0.60(0.56 to 0.65) | 6(4 to 9) | 67.96(42.66 to 100.24) | 23(14 to 34) | 81.02(50.44 to 118.43) | 0.58(0.52 to 0.64) |
| **Angola** | 164(98 to 255) | 29.41(17.59 to 45.93) | 678(414 to 1050) | 41.62(25.39 to 64.60) | 1.11(1.08 to 1.15) | 129(102 to 163) | 28.67(22.78 to 36.46) | 522(412 to 668) | 39.39(31.03 to 50.44) | 1.01(0.95 to 1.06) | 13(7 to 21) | 2.83(1.64 to 4.44) | 53(30 to 83) | 3.89(2.24 to 6.07) | 1.01(0.93 to 1.08) |
| **Antigua and Barbuda** | 2(1 to 4) | 24.41(14.62 to 38.07) | 6(4 to 9) | 35.75(21.42 to 54.98) | 1.24(1.18 to 1.30) | 2(2 to 3) | 25.04(19.69 to 32.35) | 6(4 to 7) | 35.41(28.26 to 44.93) | 1.12(1.09 to 1.15) | 0(0 to 0) | 2.50(1.39 to 4.07) | 1(0 to 1) | 3.51(1.95 to 5.60) | 1.10(1.03 to 1.18) |
| **Argentina** | 5226(3198 to 7858) | 97.88(59.75 to 147.42) | 8399(5155 to 12629) | 87.52(53.70 to 131.70) | -0.36(-0.39 to -0.33) | 9173(7468 to 11343) | 178.03(144.28 to 221.22) | 15868(12868 to 19475) | 163.17(132.49 to 200.07) | -0.28(-0.30 to -0.25) | 915(565 to 1359) | 17.66(10.90 to 26.22) | 1569(967 to 2346) | 16.16(9.94 to 24.16) | -0.28(-0.34 to -0.23) |
| **Armenia** | 247(152 to 379) | 52.11(32.09 to 79.98) | 231(140 to 354) | 30.11(18.29 to 46.29) | -1.76(-1.89 to -1.64) | 273(226 to 332) | 62.76(51.81 to 76.67) | 258(209 to 318) | 33.95(27.52 to 41.92) | -1.99(-2.18 to -1.80) | 27(16 to 42) | 6.26(3.77 to 9.62) | 26(15 to 40) | 3.39(1.93 to 5.31) | -1.96(-2.11 to -1.80) |
| **Australia** | 7408(4517 to 11092) | 226.15(137.45 to 339.32) | 28522(17565 to 42508) | 338.75(208.92 to 504.08) | 1.32(1.26 to 1.39) | 12026(9459 to 15364) | 376.86(296.10 to 482.65) | 44416(34504 to 57891) | 510.82(397.98 to 662.85) | 0.98(0.92 to 1.04) | 1182(739 to 1739) | 36.91(23.03 to 54.27) | 4308(2679 to 6390) | 49.85(31.00 to 73.96) | 0.97(0.91 to 1.03) |
| **Austria** | 6991(4316 to 10416) | 335.96(207.65 to 501.54) | 10422(6503 to 15471) | 315.15(197.34 to 466.79) | -0.19(-0.25 to -0.14) | 12355(9802 to 15462) | 598.52(476.95 to 747.45) | 18439(14592 to 23298) | 528.56(420.87 to 663.01) | -0.39(-0.44 to -0.34) | 1217(760 to 1773) | 58.98(36.85 to 85.79) | 1801(1130 to 2669) | 52.10(32.71 to 77.28) | -0.39(-0.46 to -0.31) |
| **Azerbaijan** | 163(97 to 256) | 18.46(10.98 to 29.01) | 374(225 to 587) | 20.92(12.58 to 32.80) | 0.42(0.37 to 0.48) | 210(176 to 253) | 25.96(21.66 to 31.27) | 442(364 to 534) | 27.34(22.44 to 33.22) | 0.19(0.12 to 0.25) | 21(12 to 35) | 2.62(1.45 to 4.21) | 45(24 to 73) | 2.73(1.48 to 4.43) | 0.14(0.05 to 0.24) |
| **Bahamas** | 7(4 to 12) | 31.94(19.19 to 49.77) | 25(16 to 39) | 40.31(24.59 to 61.84) | 0.77(0.70 to 0.85) | 7(6 to 9) | 32.39(25.75 to 41.10) | 24(19 to 31) | 40.86(32.52 to 51.67) | 0.75(0.62 to 0.87) | 1(0 to 1) | 3.25(1.85 to 5.14) | 2(1 to 4) | 4.07(2.35 to 6.37) | 0.73(0.62 to 0.83) |
| **Bahrain** | 7(4 to 10) | 27.54(16.42 to 43.15) | 37(22 to 57) | 32.03(19.46 to 49.67) | 0.49(0.44 to 0.53) | 6(5 to 8) | 31.03(24.94 to 38.71) | 39(32 to 47) | 37.01(29.93 to 46.00) | 0.57(0.51 to 0.64) | 1(0 to 1) | 3.08(1.75 to 4.94) | 4(2 to 6) | 3.60(1.99 to 5.77) | 0.51(0.44 to 0.58) |
| **Bangladesh** | 871(512 to 1373) | 11.75(6.92 to 18.47) | 4094(2468 to 6394) | 18.31(11.03 to 28.57) | 1.47(1.33 to 1.61) | 1034(845 to 1261) | 14.91(12.17 to 18.22) | 4623(3784 to 5656) | 21.83(17.86 to 26.75) | 1.25(1.17 to 1.34) | 104(49 to 181) | 1.48(0.71 to 2.56) | 462(231 to 776) | 2.17(1.10 to 3.60) | 1.25(1.05 to 1.45) |
| **Barbados** | 14(8 to 21) | 26.08(15.39 to 41.40) | 35(21 to 54) | 38.37(22.98 to 59.86) | 1.24(1.21 to 1.28) | 14(11 to 18) | 26.75(21.05 to 34.16) | 34(27 to 43) | 38.01(30.18 to 48.18) | 1.13(1.08 to 1.17) | 1(1 to 2) | 2.68(1.45 to 4.35) | 3(2 to 5) | 3.77(2.11 to 6.01) | 1.09(0.98 to 1.21) |
| **Belarus** | 1334(819 to 2047) | 56.65(34.82 to 87.03) | 2924(1822 to 4414) | 101.68(63.30 to 153.61) | 1.95(1.78 to 2.12) | 1843(1562 to 2188) | 81.43(68.97 to 96.72) | 3550(2974 to 4251) | 123.96(103.90 to 148.47) | 1.40(1.27 to 1.54) | 185(112 to 277) | 8.13(4.95 to 12.21) | 355(217 to 532) | 12.39(7.58 to 18.58) | 1.40(1.20 to 1.59) |
| **Belgium** | 8110(4988 to 12177) | 298.53(183.44 to 449.02) | 18174(11189 to 27113) | 431.78(266.90 to 642.70) | 1.21(1.15 to 1.27) | 14291(11400 to 17960) | 531.45(424.93 to 668.11) | 32559(25686 to 40838) | 718.67(568.70 to 896.64) | 1.00(0.93 to 1.06) | 1408(891 to 2066) | 52.30(33.14 to 76.67) | 3166(1993 to 4631) | 70.67(44.46 to 103.73) | 0.99(0.93 to 1.06) |
| **Belize** | 3(2 to 5) | 22.77(13.73 to 35.50) | 16(10 to 24) | 33.98(20.99 to 52.02) | 1.30(1.18 to 1.41) | 4(3 to 5) | 23.81(18.96 to 30.12) | 16(13 to 19) | 35.32(28.36 to 44.26) | 1.26(1.17 to 1.36) | 0(0 to 1) | 2.39(1.33 to 3.81) | 2(1 to 2) | 3.54(2.04 to 5.52) | 1.25(1.10 to 1.39) |
| **Benin** | 114(68 to 177) | 36.62(21.75 to 57.12) | 326(197 to 507) | 43.44(26.26 to 67.64) | 0.55(0.50 to 0.59) | 100(79 to 128) | 35.08(27.63 to 44.83) | 273(216 to 349) | 41.00(32.35 to 52.38) | 0.50(0.45 to 0.55) | 10(6 to 16) | 3.48(1.95 to 5.48) | 27(15 to 44) | 4.07(2.31 to 6.40) | 0.51(0.44 to 0.59) |
| **Bermuda** | 4(2 to 6) | 38.07(23.18 to 58.90) | 12(8 to 19) | 49.36(29.87 to 76.00) | 0.83(0.78 to 0.87) | 4(3 to 5) | 37.14(29.44 to 47.40) | 12(10 to 16) | 47.74(37.89 to 60.01) | 0.80(0.77 to 0.84) | 0(0 to 1) | 3.71(2.09 to 5.90) | 1(1 to 2) | 4.75(2.71 to 7.52) | 0.79(0.70 to 0.89) |
| **Bhutan** | 17(10 to 26) | 48.53(28.78 to 76.40) | 115(69 to 181) | 120.54(71.91 to 189.16) | 2.99(2.94 to 3.04) | 13(11 to 17) | 47.21(37.21 to 59.86) | 100(77 to 128) | 109.81(84.97 to 141.38) | 2.76(2.73 to 2.80) | 1(1 to 2) | 4.69(2.65 to 7.45) | 10(6 to 15) | 10.86(6.48 to 16.57) | 2.75(2.69 to 2.81) |
| **Bolivia (Plurinational State of)** | 174(105 to 272) | 33.97(20.47 to 52.99) | 737(448 to 1139) | 49.83(30.27 to 77.10) | 1.24(1.18 to 1.31) | 172(140 to 214) | 36.95(30.00 to 45.96) | 692(560 to 861) | 50.92(41.19 to 63.57) | 1.04(0.99 to 1.10) | 17(10 to 27) | 3.69(2.13 to 5.74) | 69(41 to 108) | 5.06(3.01 to 7.85) | 1.02(0.92 to 1.11) |
| **Bosnia and Herzegovina** | 498(298 to 777) | 73.77(43.97 to 115.65) | 841(515 to 1287) | 76.40(46.72 to 116.93) | 0.20(-0.11 to 0.51) | 482(392 to 595) | 79.11(64.09 to 97.82) | 903(741 to 1106) | 82.36(67.67 to 100.95) | 0.20(-0.03 to 0.42) | 49(30 to 75) | 7.88(4.82 to 12.00) | 89(54 to 135) | 8.14(4.97 to 12.28) | 0.17(-0.06 to 0.39) |
| **Botswana** | 23(14 to 37) | 27.35(16.21 to 43.18) | 69(42 to 108) | 31.48(19.00 to 49.08) | 0.44(0.39 to 0.50) | 21(17 to 26) | 28.72(23.05 to 36.10) | 63(51 to 79) | 32.52(25.94 to 40.93) | 0.40(0.33 to 0.46) | 2(1 to 3) | 2.85(1.57 to 4.58) | 6(3 to 10) | 3.20(1.79 to 5.11) | 0.37(0.27 to 0.48) |
| **Brazil** | 11081(5948 to 18637) | 79.08(42.40 to 133.10) | 36369(20571 to 59090) | 85.99(48.64 to 139.69) | 0.28(0.26 to 0.31) | 12070(9900 to 14743) | 92.61(75.66 to 113.64) | 38680(31509 to 47852) | 93.25(75.89 to 115.45) | 0.02(-0.02 to 0.06) | 1200(776 to 1740) | 9.11(5.87 to 13.22) | 3819(2429 to 5580) | 9.18(5.84 to 13.41) | 0.02(0.00 to 0.04) |
| **Brunei Darussalam** | 18(11 to 28) | 122.93(75.38 to 184.52) | 75(47 to 112) | 145.31(89.87 to 217.64) | 0.55(0.49 to 0.61) | 33(28 to 41) | 239.19(196.29 to 292.07) | 122(101 to 150) | 264.44(214.59 to 327.27) | 0.34(0.31 to 0.37) | 3(2 to 5) | 23.60(15.05 to 34.02) | 12(8 to 18) | 25.97(16.42 to 38.06) | 0.32(0.29 to 0.35) |
| **Bulgaria** | 1466(903 to 2241) | 64.39(39.69 to 98.50) | 1553(962 to 2352) | 64.76(40.03 to 98.16) | 0.09(-0.02 to 0.20) | 1833(1536 to 2168) | 86.47(72.45 to 102.41) | 2030(1694 to 2425) | 81.21(67.70 to 96.98) | -0.18(-0.29 to -0.08) | 184(115 to 273) | 8.60(5.40 to 12.76) | 202(123 to 306) | 8.09(4.89 to 12.26) | -0.22(-0.30 to -0.15) |
| **Burkina Faso** | 270(163 to 425) | 41.09(24.70 to 64.63) | 629(381 to 976) | 45.97(27.81 to 71.43) | 0.35(0.32 to 0.39) | 214(170 to 274) | 38.72(30.66 to 49.49) | 514(406 to 654) | 43.09(33.95 to 54.81) | 0.34(0.32 to 0.36) | 22(12 to 35) | 3.83(2.22 to 5.99) | 52(30 to 81) | 4.28(2.51 to 6.66) | 0.36(0.30 to 0.41) |
| **Burundi** | 127(75 to 201) | 35.50(21.06 to 56.07) | 245(149 to 375) | 35.16(21.46 to 53.80) | -0.03(-0.07 to 0.01) | 107(84 to 136) | 34.31(27.14 to 43.99) | 198(156 to 253) | 33.81(26.45 to 43.23) | -0.04(-0.09 to 0.01) | 11(6 to 17) | 3.41(1.92 to 5.47) | 20(11 to 32) | 3.38(1.96 to 5.28) | -0.03(-0.12 to 0.06) |
| **Cabo Verde** | 13(8 to 20) | 31.61(18.90 to 49.74) | 38(23 to 59) | 53.54(32.35 to 82.45) | 1.72(1.67 to 1.76) | 13(10 to 17) | 32.19(25.48 to 40.97) | 35(28 to 45) | 50.14(39.91 to 64.00) | 1.44(1.38 to 1.51) | 1(1 to 2) | 3.21(1.77 to 5.15) | 3(2 to 5) | 4.99(2.90 to 7.76) | 1.42(1.36 to 1.49) |
| **Cambodia** | 368(218 to 574) | 53.98(31.88 to 84.70) | 2042(1240 to 3178) | 106.28(64.44 to 165.56) | 2.22(2.17 to 2.26) | 308(245 to 389) | 53.11(42.20 to 67.04) | 1641(1304 to 2076) | 100.27(79.57 to 126.73) | 2.08(2.05 to 2.12) | 31(18 to 49) | 5.26(3.10 to 8.12) | 165(99 to 251) | 9.89(6.00 to 14.89) | 2.07(1.98 to 2.16) |
| **Cameroon** | 280(167 to 440) | 42.47(25.36 to 66.67) | 897(545 to 1391) | 50.39(30.63 to 78.18) | 0.53(0.48 to 0.59) | 226(179 to 288) | 40.04(31.79 to 51.20) | 720(571 to 917) | 47.34(37.45 to 60.46) | 0.53(0.48 to 0.57) | 23(13 to 36) | 3.94(2.30 to 6.19) | 73(40 to 116) | 4.70(2.66 to 7.38) | 0.55(0.46 to 0.64) |
| **Canada** | 13890(8563 to 20618) | 253.91(156.27 to 377.79) | 45091(28030 to 67174) | 341.65(212.72 to 508.21) | 0.96(0.90 to 1.03) | 21665(16973 to 27619) | 398.45(312.09 to 508.48) | 69244(53817 to 88985) | 504.43(392.14 to 647.23) | 0.76(0.69 to 0.82) | 2144(1337 to 3154) | 39.37(24.55 to 57.88) | 6751(4178 to 10028) | 49.42(30.58 to 73.53) | 0.74(0.69 to 0.78) |
| **Central African Republic** | 52(31 to 80) | 30.41(18.18 to 47.33) | 91(55 to 141) | 29.51(17.77 to 45.84) | -0.12(-0.19 to -0.05) | 39(31 to 50) | 29.24(23.02 to 37.51) | 67(54 to 86) | 28.06(22.33 to 35.64) | -0.15(-0.20 to -0.09) | 4(2 to 6) | 2.86(1.63 to 4.49) | 7(4 to 11) | 2.76(1.60 to 4.39) | -0.13(-0.19 to -0.08) |
| **Chad** | 140(83 to 220) | 31.21(18.51 to 49.03) | 303(182 to 471) | 35.92(21.59 to 55.85) | 0.45(0.41 to 0.49) | 121(95 to 156) | 30.08(23.51 to 38.67) | 248(197 to 318) | 34.20(27.04 to 43.83) | 0.41(0.38 to 0.43) | 12(7 to 20) | 2.97(1.64 to 4.76) | 25(14 to 40) | 3.40(1.94 to 5.38) | 0.43(0.35 to 0.51) |
| **Chile** | 2242(1383 to 3331) | 138.12(85.01 to 205.53) | 8454(5226 to 12711) | 188.38(116.48 to 283.08) | 0.99(0.88 to 1.10) | 3973(3239 to 4853) | 255.18(207.45 to 312.77) | 14638(11789 to 18413) | 325.75(262.30 to 409.72) | 0.79(0.71 to 0.87) | 396(249 to 582) | 25.28(15.90 to 37.11) | 1436(904 to 2097) | 31.96(20.11 to 46.68) | 0.76(0.66 to 0.86) |
| **China** | 81449(43043 to 136914) | 63.09(33.29 to 106.21) | 355855(198882 to 579705) | 102.92(57.60 to 167.76) | 1.68(1.43 to 1.93) | 74245(59398 to 93347) | 66.74(53.19 to 84.20) | 333128(265631 to 419352) | 102.74(81.57 to 129.94) | 1.48(1.21 to 1.76) | 7535(4816 to 11015) | 6.65(4.23 to 9.72) | 33271(21121 to 48695) | 10.15(6.42 to 14.84) | 1.46(1.19 to 1.73) |
| **Colombia** | 1624(998 to 2500) | 58.54(35.96 to 90.00) | 3837(2336 to 5928) | 40.43(24.64 to 62.40) | -1.20(-1.28 to -1.12) | 1609(1315 to 1979) | 62.38(50.95 to 76.81) | 4226(3431 to 5245) | 43.97(35.71 to 54.59) | -1.14(-1.21 to -1.07) | 161(97 to 244) | 6.19(3.75 to 9.35) | 422(241 to 675) | 4.40(2.52 to 7.04) | -1.10(-1.24 to -0.97) |
| **Comoros** | 8(5 to 12) | 26.78(15.83 to 42.33) | 24(14 to 37) | 32.65(19.60 to 50.51) | 0.63(0.59 to 0.68) | 6(5 to 8) | 26.07(20.56 to 33.40) | 20(16 to 26) | 31.11(24.56 to 39.83) | 0.56(0.53 to 0.59) | 1(0 to 1) | 2.57(1.45 to 4.14) | 2(1 to 3) | 3.08(1.72 to 4.90) | 0.56(0.48 to 0.65) |
| **Congo** | 60(36 to 93) | 36.86(21.99 to 57.45) | 151(91 to 234) | 40.76(24.61 to 63.32) | 0.30(0.22 to 0.37) | 46(36 to 59) | 35.12(27.65 to 45.06) | 117(93 to 149) | 38.28(30.30 to 48.53) | 0.26(0.20 to 0.32) | 5(3 to 8) | 3.48(1.95 to 5.57) | 12(7 to 19) | 3.78(2.14 to 5.94) | 0.25(0.17 to 0.33) |
| **Cook Islands** | 1(0 to 1) | 34.13(20.43 to 52.88) | 2(1 to 3) | 46.98(28.68 to 72.73) | 1.03(1.01 to 1.05) | 1(1 to 1) | 35.43(28.64 to 44.13) | 2(2 to 3) | 48.93(39.47 to 61.05) | 1.03(0.98 to 1.07) | 0(0 to 0) | 3.53(2.04 to 5.59) | 0(0 to 0) | 4.83(2.82 to 7.68) | 0.98(0.91 to 1.05) |
| **Costa Rica** | 268(164 to 414) | 97.47(59.68 to 150.68) | 816(499 to 1255) | 86.78(53.18 to 133.32) | -0.38(-0.41 to -0.34) | 251(201 to 317) | 93.57(74.73 to 118.25) | 812(649 to 1027) | 84.12(67.15 to 106.32) | -0.35(-0.37 to -0.33) | 25(15 to 38) | 9.23(5.62 to 14.15) | 80(47 to 122) | 8.32(4.92 to 12.71) | -0.35(-0.40 to -0.30) |
| **Coted'Ivoire** | 215(130 to 336) | 39.25(23.66 to 61.39) | 716(437 to 1098) | 46.63(28.45 to 71.66) | 0.55(0.51 to 0.59) | 166(131 to 213) | 37.19(29.41 to 47.64) | 569(453 to 725) | 43.71(34.77 to 55.79) | 0.51(0.46 to 0.56) | 17(9 to 26) | 3.64(2.08 to 5.68) | 57(32 to 91) | 4.33(2.49 to 6.76) | 0.54(0.47 to 0.60) |
| **Croatia** | 2100(1271 to 3201) | 213.80(129.25 to 326.37) | 4894(3015 to 7452) | 290.68(179.05 to 442.91) | 1.04(0.90 to 1.17) | 1859(1437 to 2381) | 204.21(157.57 to 262.36) | 4493(3443 to 5792) | 264.94(204.05 to 341.09) | 0.87(0.76 to 0.98) | 185(112 to 275) | 20.14(12.27 to 29.88) | 440(270 to 655) | 25.95(15.94 to 38.66) | 0.85(0.74 to 0.96) |
| **Cuba** | 1982(1207 to 3029) | 115.59(70.32 to 176.93) | 6590(4058 to 10157) | 186.92(115.16 to 287.88) | 1.56(1.52 to 1.60) | 1704(1315 to 2211) | 105.63(81.64 to 136.87) | 6145(4752 to 7992) | 167.69(129.62 to 218.01) | 1.50(1.46 to 1.53) | 171(101 to 267) | 10.54(6.25 to 16.31) | 603(360 to 908) | 16.54(9.85 to 24.96) | 1.46(1.41 to 1.51) |
| **Cyprus** | 363(219 to 546) | 296.70(177.54 to 449.73) | 1038(633 to 1571) | 292.80(177.81 to 444.70) | -0.05(-0.13 to 0.02) | 529(417 to 667) | 479.90(374.22 to 614.75) | 1577(1232 to 2003) | 475.67(372.26 to 606.12) | -0.04(-0.12 to 0.03) | 53(33 to 78) | 46.95(29.44 to 69.15) | 156(97 to 230) | 46.62(28.92 to 68.76) | -0.04(-0.12 to 0.05) |
| **Czechia** | 8142(4980 to 12525) | 340.70(207.82 to 524.91) | 7260(4490 to 11100) | 187.93(116.20 to 287.04) | -1.91(-2.03 to -1.80) | 7176(5461 to 9322) | 319.09(243.43 to 413.92) | 7014(5544 to 8846) | 178.15(141.05 to 224.36) | -1.84(-2.04 to -1.64) | 702(440 to 1054) | 30.99(19.41 to 46.36) | 688(426 to 1021) | 17.54(10.80 to 26.07) | -1.81(-2.02 to -1.61) |
| **Democratic People's Republic of Korea** | 837(495 to 1311) | 33.24(19.64 to 52.21) | 1176(712 to 1813) | 21.57(13.02 to 33.30) | -1.38(-1.41 to -1.35) | 758(603 to 949) | 34.58(27.41 to 43.34) | 1138(913 to 1440) | 22.43(17.93 to 28.45) | -1.38(-1.41 to -1.34) | 77(44 to 123) | 3.48(1.97 to 5.52) | 115(64 to 184) | 2.25(1.24 to 3.59) | -1.38(-1.44 to -1.32) |
| **Democratic Republic of the Congo** | 755(453 to 1186) | 32.48(19.44 to 51.05) | 2042(1239 to 3151) | 39.60(23.96 to 61.50) | 0.63(0.56 to 0.70) | 573(451 to 736) | 31.07(24.48 to 39.88) | 1593(1253 to 2059) | 37.49(29.43 to 48.50) | 0.60(0.53 to 0.67) | 57(32 to 94) | 3.03(1.71 to 4.87) | 161(87 to 256) | 3.71(2.09 to 5.84) | 0.64(0.50 to 0.78) |
| **Denmark** | 5953(3656 to 8908) | 403.07(247.55 to 603.55) | 5325(3259 to 7992) | 240.51(147.20 to 360.74) | -1.67(-1.73 to -1.61) | 9134(7037 to 11930) | 618.38(478.62 to 803.70) | 8740(6819 to 11238) | 386.76(302.87 to 494.93) | -1.52(-1.58 to -1.46) | 894(550 to 1333) | 60.57(37.36 to 90.03) | 860(526 to 1283) | 38.23(23.32 to 57.14) | -1.49(-1.56 to -1.43) |
| **Djibouti** | 5(3 to 8) | 29.20(17.15 to 46.03) | 32(19 to 50) | 37.32(22.50 to 57.79) | 0.79(0.77 to 0.81) | 4(3 to 5) | 28.40(22.15 to 36.71) | 25(19 to 32) | 35.41(27.81 to 45.51) | 0.71(0.69 to 0.73) | 0(0 to 1) | 2.81(1.56 to 4.53) | 2(1 to 4) | 3.51(1.99 to 5.59) | 0.71(0.63 to 0.80) |
| **Dominica** | 2(1 to 3) | 22.01(13.02 to 34.67) | 4(2 to 6) | 27.74(16.82 to 43.03) | 0.75(0.69 to 0.81) | 2(2 to 3) | 22.85(18.14 to 29.18) | 4(3 to 5) | 28.35(22.75 to 35.75) | 0.69(0.64 to 0.73) | 0(0 to 0) | 2.29(1.24 to 3.75) | 0(0 to 1) | 2.83(1.58 to 4.56) | 0.67(0.55 to 0.79) |
| **Dominican Republic** | 120(71 to 189) | 21.74(12.82 to 34.17) | 530(320 to 819) | 32.38(19.54 to 50.04) | 1.34(1.16 to 1.51) | 116(93 to 144) | 22.46(18.00 to 28.13) | 531(426 to 670) | 32.80(26.30 to 41.41) | 1.23(1.05 to 1.41) | 12(6 to 19) | 2.25(1.22 to 3.60) | 53(30 to 83) | 3.26(1.87 to 5.14) | 1.20(1.01 to 1.38) |
| **Ecuador** | 315(195 to 480) | 37.21(23.05 to 56.83) | 1384(856 to 2110) | 51.05(31.55 to 77.91) | 1.02(0.96 to 1.08) | 346(286 to 419) | 42.90(35.36 to 52.03) | 1476(1213 to 1800) | 55.80(45.82 to 68.19) | 0.86(0.79 to 0.92) | 35(20 to 55) | 4.30(2.53 to 6.71) | 148(87 to 231) | 5.57(3.27 to 8.68) | 0.84(0.75 to 0.94) |
| **Egypt** | 682(406 to 1080) | 16.92(10.03 to 26.81) | 2417(1431 to 3782) | 25.10(14.88 to 39.25) | 1.29(1.20 to 1.37) | 708(575 to 868) | 19.83(16.04 to 24.49) | 2289(1853 to 2818) | 26.67(21.60 to 33.01) | 0.96(0.93 to 0.98) | 72(41 to 114) | 1.98(1.14 to 3.11) | 231(135 to 358) | 2.64(1.57 to 4.06) | 0.93(0.85 to 1.01) |
| **El Salvador** | 242(147 to 374) | 50.34(30.64 to 77.80) | 693(423 to 1070) | 65.52(40.07 to 100.96) | 0.87(0.78 to 0.97) | 253(205 to 316) | 53.51(43.16 to 66.72) | 744(596 to 929) | 66.28(53.19 to 82.74) | 0.71(0.65 to 0.78) | 25(15 to 39) | 5.33(3.22 to 8.18) | 73(44 to 110) | 6.57(3.97 to 9.93) | 0.70(0.62 to 0.78) |
| **Equatorial Guinea** | 8(5 to 13) | 28.13(16.77 to 44.16) | 31(19 to 48) | 46.10(27.83 to 71.02) | 1.59(1.51 to 1.67) | 7(5 to 8) | 27.37(21.67 to 34.77) | 25(19 to 32) | 42.84(33.57 to 54.94) | 1.43(1.39 to 1.47) | 1(0 to 1) | 2.68(1.49 to 4.24) | 2(1 to 4) | 4.20(2.36 to 6.65) | 1.44(1.39 to 1.49) |
| **Eritrea** | 40(24 to 64) | 27.22(15.99 to 43.46) | 133(81 to 205) | 36.20(21.79 to 55.94) | 0.91(0.87 to 0.96) | 30(23 to 38) | 26.46(20.67 to 34.04) | 103(81 to 132) | 34.47(26.94 to 44.49) | 0.86(0.82 to 0.90) | 3(2 to 5) | 2.60(1.53 to 4.11) | 10(6 to 16) | 3.40(2.03 to 5.35) | 0.87(0.81 to 0.92) |
| **Estonia** | 419(255 to 637) | 116.46(71.00 to 177.33) | 389(241 to 590) | 89.05(55.15 to 135.01) | -0.83(-1.02 to -0.65) | 498(412 to 602) | 142.18(117.55 to 171.99) | 481(398 to 581) | 100.04(82.76 to 120.42) | -1.11(-1.31 to -0.92) | 50(31 to 74) | 14.21(8.93 to 21.03) | 48(29 to 72) | 10.01(6.03 to 15.16) | -1.10(-1.32 to -0.89) |
| **Eswatini** | 9(6 to 15) | 22.27(13.38 to 34.89) | 19(12 to 29) | 23.46(14.12 to 36.39) | 0.16(0.13 to 0.20) | 8(7 to 11) | 22.96(18.29 to 29.08) | 17(14 to 21) | 24.53(19.65 to 30.71) | 0.21(0.15 to 0.27) | 1(0 to 1) | 2.29(1.30 to 3.66) | 2(1 to 3) | 2.42(1.32 to 3.83) | 0.18(0.09 to 0.26) |
| **Ethiopia** | 1153(616 to 1932) | 39.18(20.97 to 65.61) | 2615(1465 to 4261) | 41.44(23.24 to 67.49) | 0.18(0.16 to 0.20) | 890(689 to 1158) | 38.23(29.65 to 49.70) | 2164(1672 to 2799) | 38.51(29.79 to 49.76) | 0.01(-0.02 to 0.05) | 89(55 to 136) | 3.74(2.32 to 5.64) | 216(130 to 329) | 3.79(2.31 to 5.74) | 0.03(-0.01 to 0.06) |
| **Fiji** | 11(7 to 18) | 23.13(13.67 to 36.22) | 32(19 to 50) | 28.34(17.06 to 43.91) | 0.65(0.59 to 0.71) | 11(9 to 13) | 25.16(20.30 to 31.67) | 29(23 to 36) | 29.48(23.52 to 36.99) | 0.51(0.47 to 0.56) | 1(1 to 2) | 2.47(1.35 to 3.99) | 3(1 to 5) | 2.87(1.55 to 4.66) | 0.48(0.37 to 0.60) |
| **Finland** | 4579(2835 to 6832) | 374.41(231.84 to 559.43) | 9187(5834 to 13617) | 407.56(259.42 to 602.10) | 0.28(0.21 to 0.34) | 8148(6448 to 10236) | 670.30(530.83 to 841.27) | 16635(13143 to 20994) | 683.82(542.36 to 857.30) | 0.07(-0.05 to 0.18) | 799(505 to 1166) | 65.68(41.52 to 95.77) | 1621(1020 to 2368) | 67.30(42.38 to 98.34) | 0.08(-0.02 to 0.18) |
| **France** | 55752(33860 to 83718) | 370.59(225.35 to 557.45) | 96960(58812 to 145866) | 367.34(224.54 to 550.03) | -0.02(-0.04 to 0.01) | 92507(72486 to 118244) | 619.98(488.24 to 789.40) | 173484(135880 to 221372) | 611.15(482.22 to 772.52) | -0.05(-0.08 to -0.02) | 9033(5646 to 13260) | 60.61(37.93 to 88.88) | 16840(10504 to 24963) | 60.18(37.52 to 89.34) | -0.02(-0.06 to 0.02) |
| **Gabon** | 42(25 to 65) | 45.32(27.06 to 70.45) | 81(50 to 124) | 52.57(32.26 to 81.12) | 0.47(0.45 to 0.49) | 35(28 to 45) | 43.00(33.99 to 54.90) | 64(51 to 82) | 48.79(38.55 to 62.15) | 0.38(0.35 to 0.41) | 4(2 to 6) | 4.24(2.46 to 6.70) | 6(4 to 10) | 4.81(2.79 to 7.45) | 0.37(0.33 to 0.41) |
| **Gambia** | 19(12 to 30) | 38.34(23.07 to 60.44) | 76(46 to 118) | 53.52(32.34 to 83.09) | 1.07(1.06 to 1.09) | 15(12 to 20) | 36.26(28.43 to 46.87) | 63(50 to 80) | 49.50(39.12 to 63.33) | 1.00(0.98 to 1.02) | 2(1 to 3) | 3.59(2.05 to 5.74) | 6(4 to 10) | 4.89(2.81 to 7.72) | 0.99(0.95 to 1.04) |
| **Georgia** | 438(268 to 670) | 39.24(24.00 to 60.00) | 651(403 to 985) | 62.35(38.55 to 94.30) | 1.53(1.37 to 1.69) | 584(489 to 697) | 54.77(45.77 to 65.61) | 840(697 to 1014) | 77.51(64.38 to 93.52) | 1.15(1.01 to 1.29) | 59(34 to 90) | 5.51(3.17 to 8.38) | 83(50 to 127) | 7.70(4.63 to 11.75) | 1.14(0.96 to 1.33) |
| **Germany** | 57698(35406 to 86697) | 257.97(158.30 to 387.93) | 106482(66720 to 158506) | 288.04(180.71 to 428.90) | 0.37(0.29 to 0.44) | 100501(79307 to 127631) | 453.92(359.97 to 575.01) | 181597(141533 to 232570) | 470.99(370.49 to 597.37) | 0.12(0.08 to 0.17) | 9890(6059 to 14679) | 44.66(27.44 to 66.04) | 17667(10991 to 26165) | 46.21(28.76 to 68.48) | 0.11(0.06 to 0.16) |
| **Ghana** | 371(220 to 582) | 41.04(24.31 to 64.37) | 1225(748 to 1883) | 51.34(31.33 to 79.13) | 0.72(0.67 to 0.77) | 295(233 to 378) | 38.79(30.61 to 49.76) | 980(779 to 1242) | 48.04(38.14 to 60.88) | 0.69(0.63 to 0.75) | 30(17 to 47) | 3.84(2.20 to 6.02) | 99(56 to 157) | 4.78(2.75 to 7.51) | 0.71(0.62 to 0.80) |
| **Greece** | 4880(3003 to 7323) | 182.28(112.05 to 273.74) | 4724(2907 to 7108) | 114.12(70.28 to 171.50) | -1.50(-1.61 to -1.39) | 9580(7810 to 11692) | 366.69(298.83 to 448.19) | 10781(8831 to 13152) | 243.53(200.32 to 295.59) | -1.30(-1.40 to -1.19) | 954(603 to 1382) | 36.39(23.00 to 52.72) | 1061(670 to 1545) | 24.25(15.27 to 35.48) | -1.27(-1.37 to -1.18) |
| **Greenland** | 21(13 to 32) | 440.97(270.67 to 671.87) | 48(30 to 72) | 413.46(255.61 to 616.86) | -0.22(-0.30 to -0.14) | 30(24 to 38) | 739.16(583.81 to 936.80) | 69(55 to 87) | 684.23(536.88 to 870.22) | -0.25(-0.31 to -0.19) | 3(2 to 4) | 72.34(45.79 to 107.12) | 7(4 to 10) | 67.29(42.11 to 99.39) | -0.24(-0.32 to -0.15) |
| **Grenada** | 4(2 to 6) | 28.07(16.93 to 43.39) | 9(5 to 13) | 48.19(29.70 to 73.79) | 1.75(1.67 to 1.83) | 4(3 to 5) | 28.72(22.84 to 36.01) | 8(6 to 10) | 46.27(36.72 to 58.96) | 1.54(1.47 to 1.62) | 0(0 to 1) | 2.88(1.65 to 4.62) | 1(0 to 1) | 4.61(2.71 to 7.22) | 1.52(1.45 to 1.60) |
| **Guam** | 4(2 to 7) | 38.50(23.22 to 60.42) | 14(9 to 22) | 37.62(23.00 to 57.73) | -0.07(-0.16 to 0.01) | 4(3 to 4) | 38.46(30.85 to 48.14) | 15(12 to 19) | 39.50(31.81 to 49.17) | 0.09(-0.07 to 0.25) | 0(0 to 1) | 3.84(2.15 to 6.16) | 2(1 to 2) | 3.94(2.17 to 6.39) | 0.08(-0.02 to 0.19) |
| **Guatemala** | 328(201 to 500) | 61.70(37.84 to 93.99) | 1281(793 to 1973) | 71.75(44.40 to 110.48) | 0.48(0.44 to 0.51) | 307(249 to 378) | 65.41(53.09 to 81.15) | 1240(1012 to 1526) | 72.93(59.43 to 89.81) | 0.34(0.30 to 0.38) | 31(18 to 48) | 6.48(3.89 to 9.96) | 123(74 to 184) | 7.20(4.32 to 10.73) | 0.32(0.25 to 0.38) |
| **Guinea** | 167(99 to 261) | 31.68(18.75 to 49.62) | 322(194 to 494) | 37.88(22.88 to 58.18) | 0.58(0.56 to 0.60) | 144(114 to 184) | 30.36(24.03 to 38.82) | 276(218 to 352) | 36.14(28.53 to 46.07) | 0.56(0.54 to 0.58) | 14(8 to 23) | 3.00(1.71 to 4.76) | 28(15 to 44) | 3.59(2.00 to 5.67) | 0.57(0.48 to 0.65) |
| **Guinea-Bissau** | 22(13 to 35) | 37.45(22.30 to 58.44) | 44(27 to 68) | 44.01(26.88 to 67.81) | 0.52(0.50 to 0.53) | 18(14 to 23) | 35.91(28.39 to 45.88) | 34(27 to 43) | 41.57(32.77 to 53.07) | 0.45(0.41 to 0.50) | 2(1 to 3) | 3.55(2.04 to 5.57) | 3(2 to 5) | 4.14(2.40 to 6.44) | 0.48(0.43 to 0.53) |
| **Guyana** | 30(18 to 46) | 52.41(31.98 to 80.85) | 60(37 to 92) | 60.23(36.99 to 92.06) | 0.45(0.39 to 0.50) | 27(22 to 34) | 51.41(41.40 to 64.74) | 55(45 to 69) | 60.77(48.99 to 76.02) | 0.53(0.50 to 0.57) | 3(2 to 4) | 5.10(2.98 to 7.89) | 5(3 to 8) | 5.97(3.51 to 9.19) | 0.51(0.43 to 0.58) |
| **Haiti** | 114(67 to 181) | 25.16(14.77 to 40.09) | 269(161 to 419) | 26.71(16.09 to 41.68) | 0.20(0.09 to 0.30) | 99(78 to 125) | 25.23(19.95 to 32.29) | 235(188 to 295) | 26.63(21.13 to 33.54) | 0.17(0.04 to 0.30) | 10(6 to 16) | 2.50(1.44 to 3.95) | 24(14 to 37) | 2.63(1.55 to 4.05) | 0.17(0.04 to 0.29) |
| **Honduras** | 86(50 to 136) | 26.38(15.43 to 42.04) | 377(229 to 582) | 36.93(22.35 to 57.22) | 1.08(1.04 to 1.12) | 94(77 to 116) | 31.48(25.59 to 38.90) | 361(290 to 449) | 38.74(31.08 to 48.23) | 0.65(0.60 to 0.70) | 9(5 to 15) | 3.13(1.77 to 4.94) | 36(21 to 57) | 3.83(2.23 to 6.03) | 0.65(0.60 to 0.70) |
| **Hungary** | 9367(5749 to 14379) | 370.91(227.09 to 569.50) | 7176(4445 to 10878) | 206.21(127.44 to 312.67) | -1.85(-2.09 to -1.61) | 8220(6360 to 10557) | 350.01(270.97 to 450.30) | 6952(5430 to 8843) | 191.62(149.98 to 243.60) | -1.90(-2.08 to -1.72) | 808(500 to 1217) | 34.09(21.08 to 51.12) | 685(418 to 1026) | 18.95(11.56 to 28.44) | -1.86(-2.06 to -1.66) |
| **Iceland** | 117(72 to 176) | 235.50(145.82 to 354.66) | 231(142 to 345) | 219.49(135.79 to 328.08) | -0.22(-0.24 to -0.20) | 214(170 to 269) | 422.45(336.33 to 528.73) | 428(342 to 537) | 390.82(312.89 to 487.94) | -0.24(-0.26 to -0.22) | 21(13 to 31) | 41.95(26.32 to 61.58) | 42(27 to 62) | 38.79(24.43 to 57.37) | -0.24(-0.27 to -0.21) |
| **India** | 100104(53176 to 168781) | 151.00(80.17 to 254.42) | 313423(174525 to 512343) | 168.73(94.08 to 275.81) | 0.36(0.35 to 0.37) | 78027(59392 to 102736) | 141.72(107.50 to 187.27) | 260721(199480 to 339558) | 157.69(120.51 to 206.01) | 0.34(0.32 to 0.37) | 7710(4821 to 11439) | 13.68(8.54 to 20.29) | 25609(16154 to 38275) | 15.26(9.61 to 22.75) | 0.35(0.33 to 0.37) |
| **Indonesia** | 9196(4854 to 15482) | 63.97(33.68 to 107.69) | 23340(13070 to 38001) | 67.17(37.68 to 109.50) | 0.17(0.12 to 0.23) | 7815(6126 to 10008) | 63.86(50.09 to 81.56) | 18565(14624 to 23709) | 63.97(50.29 to 81.72) | 0.02(-0.04 to 0.08) | 786(483 to 1174) | 6.30(3.89 to 9.36) | 1870(1158 to 2813) | 6.30(3.91 to 9.40) | 0.02(-0.04 to 0.08) |
| **Iran (Islamic Republic of)** | 1607(857 to 2705) | 40.35(21.48 to 67.86) | 5075(2864 to 8296) | 42.35(23.91 to 69.21) | 0.15(0.12 to 0.19) | 1620(1313 to 2003) | 46.01(36.97 to 57.49) | 5371(4341 to 6680) | 46.56(37.54 to 58.07) | 0.04(0.01 to 0.06) | 163(105 to 239) | 4.56(2.92 to 6.66) | 532(338 to 778) | 4.58(2.91 to 6.69) | 0.02(-0.01 to 0.05) |
| **Iraq** | 295(176 to 466) | 23.59(14.05 to 37.24) | 941(571 to 1463) | 26.00(15.70 to 40.53) | 0.32(0.31 to 0.34) | 375(307 to 457) | 30.75(25.22 to 37.53) | 1054(868 to 1279) | 31.85(26.10 to 38.87) | 0.12(0.10 to 0.14) | 37(22 to 57) | 3.04(1.82 to 4.65) | 104(64 to 156) | 3.11(1.92 to 4.67) | 0.07(0.01 to 0.14) |
| **Ireland** | 1482(909 to 2201) | 214.30(131.14 to 319.42) | 2844(1762 to 4241) | 206.55(128.13 to 308.24) | -0.12(-0.18 to -0.07) | 2515(1995 to 3174) | 374.88(297.25 to 474.23) | 5230(4153 to 6594) | 373.65(297.37 to 470.07) | -0.02(-0.11 to 0.07) | 249(156 to 370) | 37.00(23.12 to 54.86) | 516(324 to 767) | 36.99(23.17 to 54.94) | 0.00(-0.09 to 0.08) |
| **Israel** | 1065(647 to 1603) | 130.36(78.92 to 196.93) | 2772(1698 to 4182) | 127.18(77.89 to 192.00) | -0.08(-0.15 to 0.00) | 1842(1482 to 2294) | 235.22(188.98 to 293.92) | 5274(4252 to 6628) | 236.45(191.34 to 295.80) | 0.02(-0.04 to 0.09) | 183(112 to 271) | 23.28(14.27 to 34.38) | 518(322 to 761) | 23.38(14.52 to 34.42) | 0.02(-0.07 to 0.10) |
| **Italy** | 57946(31187 to 95271) | 370.63(198.93 to 611.93) | 67530(38752 to 107304) | 246.96(142.37 to 391.80) | -1.31(-1.33 to -1.28) | 94091(71556 to 124012) | 621.25(473.75 to 818.35) | 125478(97929 to 161090) | 433.99(342.42 to 550.01) | -1.15(-1.18 to -1.13) | 9206(5692 to 13593) | 60.54(37.51 to 89.15) | 12195(7664 to 17906) | 42.70(27.03 to 62.46) | -1.12(-1.15 to -1.09) |
| **Jamaica** | 56(33 to 89) | 18.74(11.00 to 29.72) | 157(95 to 241) | 29.02(17.61 to 44.52) | 1.42(1.36 to 1.48) | 64(52 to 80) | 20.94(17.01 to 26.09) | 175(140 to 219) | 30.63(24.65 to 38.26) | 1.23(1.16 to 1.30) | 6(4 to 10) | 2.11(1.14 to 3.41) | 17(10 to 28) | 3.07(1.71 to 4.92) | 1.20(1.08 to 1.31) |
| **Japan** | 41472(22533 to 67969) | 143.59(77.87 to 235.81) | 79861(45153 to 127161) | 123.01(70.21 to 195.31) | -0.49(-0.55 to -0.42) | 75749(60400 to 94299) | 270.39(215.07 to 337.89) | 161246(127383 to 203963) | 218.92(174.90 to 273.29) | -0.68(-0.72 to -0.63) | 7567(4839 to 11021) | 26.88(17.15 to 39.15) | 15799(9921 to 23097) | 21.84(13.87 to 31.95) | -0.67(-0.72 to -0.62) |
| **Jordan** | 54(32 to 84) | 28.12(16.68 to 44.02) | 336(203 to 523) | 31.06(18.89 to 48.15) | 0.32(0.27 to 0.37) | 57(46 to 70) | 32.50(26.38 to 40.34) | 349(284 to 430) | 35.18(28.40 to 43.76) | 0.25(0.23 to 0.28) | 6(3 to 9) | 3.23(1.83 to 5.05) | 35(19 to 56) | 3.46(1.93 to 5.48) | 0.22(0.14 to 0.30) |
| **Kazakhstan** | 602(366 to 920) | 28.55(17.35 to 43.68) | 1335(822 to 2042) | 42.61(26.25 to 65.24) | 1.36(1.17 to 1.55) | 840(703 to 999) | 41.99(35.08 to 50.03) | 1563(1303 to 1871) | 54.52(45.34 to 65.46) | 0.89(0.73 to 1.04) | 85(49 to 132) | 4.22(2.43 to 6.57) | 156(94 to 242) | 5.41(3.25 to 8.36) | 0.85(0.64 to 1.06) |
| **Kenya** | 469(251 to 787) | 38.21(20.46 to 64.10) | 1535(862 to 2503) | 47.80(26.87 to 77.95) | 0.72(0.69 to 0.75) | 393(304 to 513) | 36.43(28.17 to 47.53) | 1211(941 to 1564) | 44.68(34.64 to 57.65) | 0.66(0.63 to 0.68) | 39(25 to 59) | 3.58(2.25 to 5.36) | 122(78 to 182) | 4.39(2.81 to 6.53) | 0.66(0.63 to 0.69) |
| **Kiribati** | 1(0 to 1) | 14.43(8.33 to 23.25) | 2(1 to 3) | 17.02(10.08 to 27.21) | 0.53(0.50 to 0.56) | 1(1 to 1) | 16.23(12.97 to 20.66) | 2(1 to 2) | 18.69(14.88 to 23.64) | 0.43(0.40 to 0.47) | 0(0 to 0) | 1.58(0.82 to 2.65) | 0(0 to 0) | 1.82(0.94 to 3.11) | 0.45(0.39 to 0.52) |
| **Kuwait** | 25(15 to 38) | 28.49(17.31 to 43.36) | 145(90 to 223) | 33.96(21.01 to 51.79) | 0.56(0.48 to 0.63) | 31(26 to 37) | 39.51(32.66 to 47.63) | 183(153 to 220) | 45.34(37.79 to 54.73) | 0.44(0.41 to 0.48) | 3(2 to 5) | 3.95(2.27 to 6.21) | 18(10 to 29) | 4.49(2.54 to 7.07) | 0.41(0.31 to 0.50) |
| **Kyrgyzstan** | 150(92 to 227) | 28.39(17.35 to 43.01) | 187(115 to 288) | 22.79(14.00 to 34.99) | -0.68(-0.80 to -0.56) | 202(170 to 241) | 40.63(34.13 to 48.61) | 223(186 to 269) | 29.80(24.75 to 36.17) | -0.98(-1.06 to -0.91) | 21(12 to 31) | 4.11(2.39 to 6.27) | 23(12 to 36) | 3.00(1.66 to 4.78) | -0.99(-1.07 to -0.90) |
| **Lao People's Democratic Republic** | 87(51 to 136) | 26.75(15.64 to 41.91) | 297(180 to 453) | 42.15(25.52 to 64.46) | 1.50(1.45 to 1.54) | 78(63 to 97) | 27.96(22.49 to 35.16) | 256(205 to 323) | 41.69(33.43 to 52.62) | 1.31(1.26 to 1.37) | 8(4 to 13) | 2.79(1.58 to 4.47) | 26(14 to 41) | 4.13(2.39 to 6.57) | 1.29(1.17 to 1.41) |
| **Latvia** | 885(552 to 1347) | 141.60(88.17 to 215.50) | 659(408 to 997) | 98.82(61.19 to 149.45) | -1.16(-1.22 to -1.09) | 1077(891 to 1301) | 175.28(144.98 to 211.81) | 798(664 to 962) | 111.02(92.52 to 133.86) | -1.48(-1.59 to -1.37) | 107(67 to 158) | 17.40(10.92 to 25.64) | 79(48 to 118) | 11.08(6.78 to 16.59) | -1.46(-1.58 to -1.35) |
| **Lebanon** | 85(50 to 137) | 26.87(15.67 to 42.96) | 365(217 to 579) | 33.88(20.11 to 53.78) | 0.75(0.68 to 0.82) | 92(74 to 116) | 30.43(24.30 to 38.32) | 413(331 to 517) | 38.10(30.64 to 47.46) | 0.73(0.66 to 0.80) | 9(5 to 14) | 2.97(1.80 to 4.55) | 40(24 to 62) | 3.71(2.21 to 5.74) | 0.72(0.66 to 0.78) |
| **Lesotho** | 28(17 to 44) | 20.92(12.33 to 33.19) | 43(26 to 66) | 25.86(15.61 to 40.30) | 0.69(0.65 to 0.73) | 27(21 to 34) | 21.77(17.26 to 27.67) | 38(30 to 47) | 26.33(21.14 to 32.73) | 0.62(0.57 to 0.67) | 3(1 to 4) | 2.18(1.17 to 3.52) | 4(2 to 6) | 2.58(1.46 to 4.10) | 0.56(0.47 to 0.64) |
| **Liberia** | 64(38 to 99) | 35.24(21.06 to 54.85) | 127(76 to 196) | 44.26(26.67 to 68.66) | 0.72(0.64 to 0.80) | 53(42 to 68) | 33.69(26.46 to 43.19) | 104(82 to 134) | 41.55(32.74 to 53.42) | 0.66(0.62 to 0.71) | 5(3 to 9) | 3.32(1.85 to 5.26) | 10(6 to 17) | 4.09(2.38 to 6.51) | 0.65(0.59 to 0.72) |
| **Libya** | 89(53 to 140) | 30.18(18.06 to 47.47) | 285(172 to 443) | 37.15(22.45 to 57.68) | 0.67(0.51 to 0.82) | 102(83 to 125) | 35.88(29.12 to 44.18) | 303(246 to 369) | 42.04(33.94 to 51.43) | 0.50(0.39 to 0.62) | 10(6 to 16) | 3.59(2.12 to 5.59) | 30(18 to 45) | 4.13(2.46 to 6.22) | 0.45(0.34 to 0.57) |
| **Lithuania** | 937(578 to 1434) | 117.43(72.41 to 179.67) | 1317(814 to 2024) | 134.28(82.94 to 206.30) | 0.44(0.27 to 0.62) | 1175(983 to 1403) | 150.02(125.65 to 179.03) | 1531(1261 to 1861) | 145.23(119.67 to 176.64) | -0.12(-0.25 to 0.01) | 118(74 to 173) | 14.98(9.43 to 22.01) | 152(94 to 225) | 14.45(8.92 to 21.56) | -0.13(-0.23 to -0.03) |
| **Luxembourg** | 279(171 to 419) | 297.01(182.04 to 445.92) | 590(366 to 882) | 309.31(192.15 to 460.78) | 0.13(0.10 to 0.17) | 487(389 to 607) | 527.85(421.58 to 657.60) | 1028(808 to 1312) | 523.15(412.66 to 664.63) | -0.03(-0.05 to 0.00) | 48(30 to 71) | 52.22(32.90 to 76.95) | 101(62 to 149) | 51.62(31.96 to 75.97) | -0.04(-0.09 to 0.02) |
| **Madagascar** | 173(103 to 273) | 22.80(13.52 to 35.87) | 406(243 to 626) | 26.49(15.83 to 40.87) | 0.48(0.47 to 0.50) | 151(120 to 190) | 22.70(18.05 to 28.59) | 323(256 to 410) | 25.82(20.39 to 32.91) | 0.41(0.39 to 0.43) | 15(8 to 25) | 2.24(1.22 to 3.62) | 33(17 to 55) | 2.56(1.41 to 4.19) | 0.42(0.35 to 0.48) |
| **Malawi** | 184(110 to 285) | 32.77(19.54 to 50.99) | 442(270 to 683) | 42.87(26.03 to 66.37) | 0.87(0.85 to 0.89) | 147(116 to 189) | 31.34(24.72 to 40.33) | 353(279 to 449) | 40.24(31.69 to 51.15) | 0.81(0.78 to 0.83) | 15(8 to 25) | 3.08(1.70 to 5.04) | 36(20 to 58) | 3.97(2.27 to 6.38) | 0.82(0.76 to 0.89) |
| **Malaysia** | 656(393 to 1023) | 46.60(27.89 to 72.71) | 2873(1744 to 4445) | 62.43(37.86 to 96.69) | 0.96(0.91 to 1.01) | 590(471 to 755) | 44.31(35.35 to 56.75) | 2421(1928 to 3065) | 58.17(46.40 to 73.62) | 0.89(0.86 to 0.92) | 58(34 to 92) | 4.36(2.53 to 6.86) | 241(141 to 374) | 5.73(3.39 to 8.82) | 0.89(0.84 to 0.94) |
| **Maldives** | 6(4 to 10) | 42.80(25.48 to 67.05) | 35(21 to 53) | 69.74(42.48 to 106.76) | 1.59(1.54 to 1.64) | 5(4 to 6) | 43.91(35.26 to 55.10) | 31(25 to 39) | 67.35(54.08 to 84.02) | 1.39(1.35 to 1.43) | 1(0 to 1) | 4.39(2.50 to 6.98) | 3(2 to 5) | 6.69(3.88 to 10.44) | 1.38(1.32 to 1.45) |
| **Mali** | 261(155 to 412) | 44.01(26.02 to 69.69) | 691(413 to 1083) | 53.51(31.96 to 83.96) | 0.63(0.56 to 0.70) | 207(163 to 265) | 42.19(33.26 to 54.13) | 546(431 to 691) | 50.02(39.54 to 62.97) | 0.55(0.47 to 0.63) | 21(12 to 33) | 4.15(2.42 to 6.51) | 55(32 to 88) | 4.95(2.90 to 7.78) | 0.56(0.47 to 0.65) |
| **Malta** | 177(108 to 268) | 249.48(152.16 to 377.57) | 370(230 to 559) | 210.46(130.68 to 317.41) | -0.54(-0.61 to -0.47) | 296(234 to 371) | 433.44(342.30 to 545.08) | 695(554 to 873) | 382.18(306.19 to 477.39) | -0.40(-0.48 to -0.31) | 30(18 to 44) | 43.02(26.64 to 63.94) | 69(43 to 101) | 37.90(23.43 to 55.96) | -0.40(-0.49 to -0.31) |
| **Marshall Islands** | 1(0 to 1) | 31.55(18.77 to 49.44) | 2(1 to 3) | 40.67(24.66 to 62.44) | 0.81(0.77 to 0.85) | 1(1 to 1) | 32.06(25.51 to 40.36) | 2(1 to 2) | 41.21(33.24 to 51.34) | 0.80(0.77 to 0.84) | 0(0 to 0) | 3.17(1.77 to 5.05) | 0(0 to 0) | 4.06(2.31 to 6.40) | 0.76(0.69 to 0.84) |
| **Mauritania** | 59(35 to 93) | 37.68(22.38 to 59.29) | 149(90 to 234) | 45.37(27.41 to 71.07) | 0.59(0.55 to 0.64) | 49(39 to 63) | 35.71(28.04 to 45.93) | 125(99 to 161) | 42.24(33.29 to 54.24) | 0.54(0.49 to 0.58) | 5(3 to 8) | 3.55(2.07 to 5.61) | 13(7 to 20) | 4.21(2.43 to 6.61) | 0.55(0.47 to 0.63) |
| **Mauritius** | 27(17 to 42) | 22.76(13.79 to 35.07) | 104(65 to 159) | 31.48(19.45 to 48.00) | 1.04(0.93 to 1.15) | 28(23 to 34) | 25.50(20.91 to 31.23) | 110(91 to 136) | 34.96(28.78 to 43.05) | 1.04(0.98 to 1.09) | 3(1 to 5) | 2.54(1.35 to 4.18) | 11(6 to 18) | 3.46(1.87 to 5.55) | 1.01(0.89 to 1.13) |
| **Mexico** | 7577(4078 to 12689) | 112.79(60.66 to 188.99) | 16455(9262 to 26814) | 79.55(44.76 to 129.59) | -1.14(-1.25 to -1.02) | 7315(5848 to 9137) | 117.23(93.69 to 146.42) | 16184(13090 to 20135) | 81.51(65.83 to 101.53) | -1.15(-1.27 to -1.04) | 728(465 to 1055) | 11.56(7.37 to 16.72) | 1608(1031 to 2350) | 8.06(5.16 to 11.78) | -1.15(-1.25 to -1.04) |
| **Micronesia (Federated States of)** | 2(1 to 4) | 31.74(18.79 to 49.84) | 5(3 to 8) | 48.37(29.21 to 74.97) | 1.36(1.31 to 1.41) | 2(2 to 3) | 32.40(25.88 to 40.92) | 4(3 to 5) | 47.89(38.01 to 60.40) | 1.25(1.20 to 1.30) | 0(0 to 0) | 3.20(1.86 to 5.11) | 0(0 to 1) | 4.70(2.73 to 7.32) | 1.23(1.17 to 1.28) |
| **Monaco** | 19(12 to 30) | 152.19(90.68 to 234.22) | 29(17 to 44) | 157.86(95.54 to 240.50) | 0.12(0.08 to 0.17) | 37(30 to 47) | 285.10(228.34 to 356.54) | 56(45 to 71) | 297.30(239.44 to 371.96) | 0.14(0.11 to 0.17) | 4(2 to 5) | 28.43(17.66 to 41.78) | 6(3 to 8) | 29.56(18.41 to 43.96) | 0.13(0.09 to 0.17) |
| **Mongolia** | 54(32 to 85) | 30.67(18.13 to 48.15) | 159(96 to 244) | 39.12(23.71 to 60.13) | 0.82(0.77 to 0.87) | 68(56 to 82) | 41.65(34.28 to 50.20) | 194(162 to 232) | 55.30(46.17 to 66.11) | 0.95(0.88 to 1.01) | 7(4 to 11) | 4.17(2.40 to 6.44) | 20(11 to 30) | 5.53(3.20 to 8.45) | 0.94(0.84 to 1.04) |
| **Montenegro** | 134(81 to 206) | 127.31(77.41 to 195.88) | 194(121 to 297) | 114.28(70.88 to 175.12) | -0.27(-0.43 to -0.12) | 137(111 to 168) | 135.15(109.83 to 166.21) | 191(156 to 234) | 118.67(96.80 to 145.68) | -0.37(-0.54 to -0.19) | 14(8 to 21) | 13.51(8.26 to 20.19) | 19(12 to 29) | 11.82(7.24 to 17.79) | -0.37(-0.55 to -0.19) |
| **Morocco** | 792(450 to 1277) | 35.95(20.39 to 58.10) | 3030(1793 to 4804) | 56.55(33.37 to 89.89) | 1.48(1.43 to 1.52) | 842(677 to 1045) | 40.71(32.58 to 50.70) | 2929(2352 to 3654) | 59.13(47.33 to 74.07) | 1.21(1.19 to 1.24) | 84(49 to 132) | 4.03(2.34 to 6.26) | 290(170 to 452) | 5.80(3.43 to 8.99) | 1.18(1.13 to 1.22) |
| **Mozambique** | 268(157 to 424) | 30.97(18.14 to 48.98) | 660(398 to 1025) | 42.83(25.73 to 66.61) | 1.06(1.04 to 1.08) | 221(175 to 283) | 30.24(23.82 to 38.87) | 522(411 to 667) | 40.34(31.56 to 51.72) | 0.95(0.91 to 0.98) | 22(12 to 36) | 2.97(1.70 to 4.70) | 52(30 to 83) | 3.95(2.31 to 6.21) | 0.95(0.91 to 1.00) |
| **Myanmar** | 1649(983 to 2577) | 45.61(27.07 to 71.51) | 5480(3314 to 8458) | 71.80(43.40 to 110.84) | 1.49(1.46 to 1.53) | 1455(1166 to 1839) | 46.57(37.33 to 59.04) | 4823(3848 to 6058) | 69.95(55.68 to 88.18) | 1.33(1.31 to 1.36) | 146(84 to 231) | 4.60(2.68 to 7.18) | 482(278 to 741) | 6.90(4.03 to 10.50) | 1.33(1.28 to 1.37) |
| **Namibia** | 21(12 to 32) | 20.71(12.38 to 32.66) | 56(34 to 87) | 27.19(16.40 to 42.17) | 0.87(0.79 to 0.95) | 18(15 to 23) | 21.44(17.12 to 27.31) | 50(40 to 63) | 27.73(22.12 to 35.12) | 0.82(0.76 to 0.89) | 2(1 to 3) | 2.14(1.19 to 3.40) | 5(3 to 8) | 2.76(1.55 to 4.34) | 0.80(0.73 to 0.88) |
| **Nauru** | 0(0 to 0) | 37.24(22.48 to 58.50) | 0(0 to 1) | 56.55(33.83 to 89.58) | 1.35(1.32 to 1.38) | 0(0 to 0) | 38.44(30.80 to 47.66) | 0(0 to 0) | 56.18(45.11 to 70.64) | 1.23(1.20 to 1.26) | 0(0 to 0) | 3.81(2.20 to 5.99) | 0(0 to 0) | 5.51(3.24 to 8.48) | 1.19(1.13 to 1.26) |
| **Nepal** | 958(549 to 1556) | 68.06(38.75 to 111.34) | 3721(2197 to 5840) | 99.21(58.30 to 156.36) | 1.24(1.17 to 1.31) | 893(721 to 1109) | 73.38(59.04 to 91.74) | 3440(2747 to 4312) | 102.23(81.30 to 128.63) | 1.08(1.02 to 1.13) | 89(50 to 139) | 7.19(4.16 to 11.08) | 341(203 to 530) | 10.03(6.00 to 15.43) | 1.08(0.97 to 1.19) |
| **Netherlands** | 7926(4798 to 12016) | 226.36(136.88 to 343.54) | 24554(15138 to 36980) | 376.22(232.31 to 566.50) | 1.64(1.28 to 2.01) | 12410(9701 to 16062) | 359.85(281.81 to 464.69) | 37102(28516 to 48342) | 556.07(428.31 to 722.39) | 1.43(1.20 to 1.67) | 1221(750 to 1806) | 35.36(21.73 to 52.24) | 3613(2237 to 5362) | 54.39(33.65 to 80.79) | 1.42(1.19 to 1.64) |
| **New Zealand** | 2073(1115 to 3408) | 312.25(167.60 to 515.01) | 4396(2511 to 6982) | 285.80(163.50 to 453.58) | -0.27(-0.36 to -0.19) | 3263(2467 to 4302) | 508.44(384.82 to 671.09) | 7044(5427 to 9169) | 452.27(349.32 to 587.05) | -0.37(-0.43 to -0.31) | 318(195 to 471) | 49.25(30.27 to 73.02) | 685(424 to 1015) | 44.12(27.32 to 65.34) | -0.35(-0.40 to -0.29) |
| **Nicaragua** | 113(68 to 175) | 48.50(29.06 to 75.12) | 556(340 to 852) | 73.90(45.15 to 113.29) | 1.36(1.30 to 1.42) | 107(85 to 133) | 48.80(38.63 to 61.09) | 496(394 to 626) | 69.80(55.33 to 88.10) | 1.17(1.11 to 1.22) | 11(6 to 16) | 4.82(2.86 to 7.41) | 49(29 to 75) | 6.85(4.08 to 10.50) | 1.14(1.07 to 1.21) |
| **Niger** | 160(93 to 256) | 41.18(23.80 to 66.19) | 624(374 to 978) | 51.37(30.72 to 80.45) | 0.71(0.66 to 0.75) | 126(99 to 163) | 39.80(31.03 to 51.32) | 482(376 to 616) | 47.89(37.35 to 61.24) | 0.59(0.51 to 0.67) | 13(7 to 20) | 3.92(2.22 to 6.21) | 49(27 to 78) | 4.76(2.73 to 7.45) | 0.62(0.57 to 0.68) |
| **Nigeria** | 2706(1448 to 4550) | 40.37(21.60 to 67.93) | 6569(3672 to 10726) | 51.11(28.61 to 83.35) | 0.76(0.72 to 0.80) | 2298(1779 to 2989) | 39.01(30.15 to 50.72) | 5343(4147 to 6878) | 47.62(36.98 to 61.21) | 0.64(0.59 to 0.68) | 229(142 to 343) | 3.83(2.39 to 5.71) | 535(334 to 802) | 4.70(2.94 to 7.02) | 0.65(0.60 to 0.70) |
| **Niue** | 0(0 to 0) | 35.55(20.95 to 56.11) | 0(0 to 0) | 48.99(29.73 to 76.47) | 1.03(0.98 to 1.08) | 0(0 to 0) | 36.31(28.88 to 45.79) | 0(0 to 0) | 47.98(38.13 to 60.45) | 0.89(0.86 to 0.92) | 0(0 to 0) | 3.59(2.01 to 5.67) | 0(0 to 0) | 4.72(2.69 to 7.44) | 0.88(0.83 to 0.94) |
| **North Macedonia** | 127(75 to 204) | 41.48(24.48 to 66.12) | 501(305 to 774) | 93.52(56.67 to 144.88) | 2.72(2.56 to 2.89) | 142(115 to 176) | 48.80(39.31 to 60.45) | 455(367 to 565) | 96.16(76.97 to 120.57) | 2.24(2.05 to 2.44) | 14(8 to 22) | 4.85(2.85 to 7.48) | 45(27 to 68) | 9.43(5.65 to 14.15) | 2.21(1.99 to 2.43) |
| **Northern Mariana Islands** | 1(1 to 2) | 62.46(37.18 to 97.11) | 7(4 to 10) | 86.55(53.44 to 132.43) | 1.05(0.89 to 1.21) | 1(1 to 1) | 61.02(48.91 to 76.48) | 5(4 to 7) | 83.11(66.86 to 104.10) | 1.00(0.95 to 1.04) | 0(0 to 0) | 6.09(3.59 to 9.52) | 1(0 to 1) | 8.22(4.83 to 12.54) | 0.98(0.89 to 1.08) |
| **Norway** | 4676(2527 to 7706) | 372.49(201.53 to 614.18) | 7223(4048 to 11602) | 382.34(215.17 to 613.16) | 0.08(0.03 to 0.12) | 7456(5588 to 9988) | 588.16(443.91 to 782.70) | 11237(8489 to 14985) | 565.80(428.57 to 751.49) | -0.13(-0.17 to -0.08) | 724(444 to 1078) | 57.28(35.35 to 85.03) | 1084(668 to 1616) | 55.13(34.02 to 82.21) | -0.13(-0.16 to -0.09) |
| **Oman** | 67(40 to 105) | 72.64(43.17 to 114.57) | 239(144 to 370) | 97.82(58.49 to 152.24) | 0.97(0.93 to 1.01) | 62(50 to 77) | 74.14(60.11 to 91.80) | 202(162 to 251) | 95.04(75.80 to 119.34) | 0.81(0.78 to 0.84) | 6(4 to 9) | 7.32(4.52 to 11.08) | 20(12 to 31) | 9.31(5.57 to 14.11) | 0.78(0.74 to 0.82) |
| **Pakistan** | 2063(1099 to 3460) | 23.48(12.52 to 39.38) | 4428(2504 to 7202) | 25.16(14.24 to 40.91) | 0.22(0.19 to 0.26) | 1966(1544 to 2554) | 24.50(19.24 to 31.89) | 3969(3128 to 5080) | 25.41(19.90 to 32.60) | 0.13(0.09 to 0.16) | 195(115 to 301) | 2.41(1.43 to 3.70) | 395(234 to 616) | 2.49(1.50 to 3.86) | 0.12(0.02 to 0.21) |
| **Palau** | 1(1 to 2) | 92.52(54.77 to 145.21) | 4(2 to 6) | 104.97(63.89 to 162.99) | 0.38(0.31 to 0.46) | 1(1 to 1) | 91.11(73.19 to 114.67) | 3(2 to 4) | 103.18(83.24 to 128.29) | 0.37(0.30 to 0.45) | 0(0 to 0) | 8.99(5.39 to 13.90) | 0(0 to 0) | 10.13(6.11 to 15.32) | 0.36(0.28 to 0.45) |
| **Palestine** | 53(31 to 83) | 38.95(23.06 to 61.41) | 179(109 to 276) | 48.95(29.64 to 75.75) | 0.75(0.69 to 0.81) | 55(44 to 69) | 43.39(34.96 to 54.46) | 175(141 to 217) | 52.88(42.34 to 66.25) | 0.65(0.59 to 0.72) | 5(3 to 9) | 4.30(2.58 to 6.67) | 17(11 to 27) | 5.22(3.16 to 8.02) | 0.63(0.54 to 0.72) |
| **Panama** | 110(67 to 170) | 46.97(28.49 to 72.26) | 264(160 to 408) | 35.20(21.37 to 54.42) | -0.93(-0.99 to -0.86) | 113(91 to 140) | 49.29(39.85 to 61.47) | 298(241 to 367) | 39.13(31.67 to 48.07) | -0.76(-0.82 to -0.70) | 11(7 to 17) | 4.90(2.90 to 7.58) | 29(17 to 46) | 3.88(2.23 to 6.07) | -0.76(-0.85 to -0.68) |
| **Papua New Guinea** | 117(65 to 197) | 55.25(30.44 to 92.67) | 469(268 to 766) | 78.05(44.68 to 127.62) | 1.11(1.05 to 1.16) | 95(74 to 122) | 55.25(42.38 to 71.61) | 384(300 to 494) | 76.60(59.03 to 99.87) | 1.04(0.99 to 1.10) | 10(5 to 16) | 5.38(3.03 to 8.46) | 38(22 to 60) | 7.46(4.28 to 11.47) | 1.04(0.96 to 1.11) |
| **Paraguay** | 143(86 to 222) | 40.56(24.24 to 62.80) | 488(294 to 755) | 51.34(31.01 to 79.47) | 0.76(0.66 to 0.86) | 174(144 to 210) | 50.44(41.47 to 60.89) | 524(426 to 645) | 56.39(45.77 to 69.55) | 0.36(0.31 to 0.40) | 17(10 to 27) | 5.02(2.89 to 7.84) | 52(31 to 82) | 5.62(3.30 to 8.77) | 0.35(0.30 to 0.40) |
| **Peru** | 483(293 to 754) | 24.83(15.07 to 38.81) | 2017(1223 to 3107) | 36.21(21.96 to 55.73) | 1.21(1.16 to 1.25) | 519(424 to 639) | 27.88(22.80 to 34.34) | 2248(1829 to 2772) | 40.31(32.80 to 49.69) | 1.19(1.13 to 1.24) | 52(30 to 84) | 2.80(1.60 to 4.47) | 226(130 to 352) | 4.05(2.33 to 6.32) | 1.18(1.11 to 1.25) |
| **Philippines** | 1764(943 to 2950) | 39.55(21.12 to 66.11) | 5341(3001 to 8712) | 41.88(23.51 to 68.37) | 0.18(0.16 to 0.20) | 1634(1307 to 2065) | 41.05(32.86 to 51.85) | 4797(3844 to 6091) | 41.70(33.39 to 53.07) | 0.06(0.02 to 0.09) | 164(104 to 243) | 4.05(2.58 to 5.99) | 481(306 to 710) | 4.13(2.63 to 6.11) | 0.07(0.03 to 0.10) |
| **Poland** | 11094(5937 to 18740) | 149.07(79.62 to 252.16) | 17449(9898 to 28480) | 139.79(79.48 to 228.44) | -0.17(-0.30 to -0.05) | 11106(8655 to 14311) | 157.34(122.29 to 203.52) | 18777(15007 to 23610) | 145.47(116.37 to 182.81) | -0.22(-0.33 to -0.11) | 1097(695 to 1604) | 15.43(9.75 to 22.56) | 1851(1173 to 2701) | 14.39(9.12 to 21.00) | -0.19(-0.30 to -0.08) |
| **Portugal** | 3550(2206 to 5305) | 148.00(91.82 to 221.70) | 7019(4320 to 10507) | 160.33(99.13 to 240.25) | 0.27(0.23 to 0.31) | 7172(5855 to 8756) | 306.31(249.37 to 375.46) | 13674(10949 to 17184) | 293.01(236.07 to 365.23) | -0.14(-0.16 to -0.12) | 712(455 to 1034) | 30.28(19.34 to 44.00) | 1346(844 to 1985) | 29.08(18.23 to 42.89) | -0.13(-0.16 to -0.10) |
| **Puerto Rico** | 306(187 to 470) | 51.34(31.39 to 78.97) | 1004(615 to 1532) | 79.96(49.04 to 121.96) | 1.44(1.39 to 1.49) | 311(252 to 388) | 53.28(43.11 to 66.53) | 1134(916 to 1415) | 79.72(64.39 to 99.46) | 1.29(1.23 to 1.35) | 31(18 to 49) | 5.35(3.10 to 8.37) | 112(66 to 170) | 7.97(4.66 to 12.20) | 1.28(1.18 to 1.38) |
| **Qatar** | 6(4 to 10) | 42.08(25.22 to 65.69) | 64(39 to 99) | 63.37(38.64 to 98.48) | 1.31(1.26 to 1.36) | 6(5 to 8) | 50.06(41.17 to 61.15) | 64(53 to 78) | 69.98(56.68 to 86.59) | 1.08(1.04 to 1.12) | 1(0 to 1) | 4.95(2.87 to 7.81) | 7(4 to 11) | 6.86(3.95 to 10.64) | 1.05(0.97 to 1.12) |
| **Republic of Korea** | 7842(4847 to 11927) | 163.04(99.76 to 248.88) | 43792(26882 to 65646) | 267.78(164.13 to 401.93) | 1.61(1.56 to 1.66) | 14940(12228 to 17983) | 344.21(279.95 to 417.71) | 72380(57379 to 91113) | 449.54(356.06 to 567.01) | 0.86(0.77 to 0.94) | 1499(960 to 2184) | 34.07(21.80 to 49.48) | 7166(4440 to 10584) | 44.38(27.49 to 65.51) | 0.86(0.78 to 0.93) |
| **Republic of Moldova** | 662(408 to 1015) | 83.52(51.47 to 128.10) | 682(420 to 1029) | 64.30(39.59 to 97.18) | -0.80(-1.01 to -0.59) | 744(618 to 903) | 103.02(85.67 to 124.78) | 765(632 to 931) | 72.67(60.04 to 88.40) | -1.11(-1.25 to -0.96) | 75(46 to 111) | 10.31(6.30 to 15.28) | 77(46 to 120) | 7.31(4.40 to 11.35) | -1.10(-1.23 to -0.96) |
| **Romania** | 3908(2411 to 5945) | 76.30(47.09 to 116.01) | 5187(3218 to 7864) | 88.46(54.78 to 134.19) | 0.53(0.45 to 0.62) | 5020(4244 to 5922) | 104.58(88.65 to 123.26) | 6596(5520 to 7909) | 102.79(85.91 to 123.50) | -0.04(-0.24 to 0.16) | 504(311 to 750) | 10.44(6.46 to 15.50) | 659(405 to 989) | 10.33(6.28 to 15.53) | -0.01(-0.20 to 0.18) |
| **Russian Federation** | 19064(10292 to 32073) | 60.09(32.33 to 101.14) | 32346(18264 to 52363) | 76.70(43.38 to 124.38) | 0.80(0.65 to 0.94) | 24684(20533 to 29572) | 81.74(67.91 to 98.07) | 41548(34711 to 49682) | 98.41(82.19 to 117.70) | 0.59(0.44 to 0.74) | 2461(1615 to 3505) | 8.11(5.32 to 11.56) | 4116(2695 to 5844) | 9.74(6.37 to 13.83) | 0.59(0.44 to 0.73) |
| **Rwanda** | 130(77 to 205) | 31.17(18.43 to 48.98) | 365(222 to 564) | 40.83(24.74 to 63.23) | 0.86(0.80 to 0.91) | 104(82 to 135) | 30.25(23.57 to 39.28) | 292(230 to 372) | 38.59(30.30 to 49.19) | 0.77(0.71 to 0.82) | 11(6 to 17) | 2.99(1.69 to 4.77) | 30(18 to 46) | 3.82(2.28 to 5.92) | 0.78(0.71 to 0.85) |
| **Saint Kitts and Nevis** | 2(1 to 3) | 33.48(20.51 to 51.51) | 5(3 to 8) | 51.62(31.70 to 79.00) | 1.41(1.37 to 1.46) | 2(2 to 3) | 33.22(26.26 to 42.14) | 5(4 to 6) | 49.28(39.09 to 62.52) | 1.27(1.23 to 1.32) | 0(0 to 0) | 3.31(1.89 to 5.20) | 0(0 to 1) | 4.89(2.78 to 7.64) | 1.27(1.19 to 1.35) |
| **Saint Lucia** | 3(2 to 5) | 24.41(14.70 to 37.71) | 12(8 to 19) | 30.30(18.63 to 46.42) | 0.71(0.62 to 0.80) | 3(3 to 4) | 25.00(19.78 to 31.54) | 12(10 to 16) | 31.46(25.25 to 39.56) | 0.74(0.67 to 0.82) | 0(0 to 1) | 2.48(1.39 to 3.97) | 1(1 to 2) | 3.11(1.76 to 4.97) | 0.74(0.62 to 0.86) |
| **Saint Vincent and the Grenadines** | 3(2 to 5) | 28.51(17.34 to 43.75) | 10(6 to 15) | 41.56(25.60 to 62.53) | 1.21(1.10 to 1.31) | 3(3 to 4) | 28.82(23.03 to 35.74) | 10(8 to 12) | 42.77(34.89 to 52.77) | 1.27(1.16 to 1.37) | 0(0 to 1) | 2.89(1.60 to 4.59) | 1(1 to 2) | 4.23(2.45 to 6.66) | 1.21(1.06 to 1.36) |
| **Samoa** | 4(3 to 7) | 34.34(20.36 to 53.74) | 10(6 to 16) | 47.81(28.94 to 73.69) | 1.07(1.03 to 1.12) | 4(3 to 5) | 34.70(27.62 to 43.95) | 10(8 to 12) | 47.49(38.15 to 59.63) | 1.02(0.96 to 1.08) | 0(0 to 1) | 3.44(1.98 to 5.55) | 1(1 to 2) | 4.68(2.70 to 7.37) | 1.00(0.92 to 1.09) |
| **San Marino** | 10(6 to 15) | 160.00(95.71 to 246.44) | 25(15 to 38) | 175.83(106.40 to 264.93) | 0.32(0.28 to 0.36) | 18(15 to 23) | 294.45(235.13 to 369.74) | 49(39 to 61) | 316.30(252.34 to 392.88) | 0.24(0.19 to 0.29) | 2(1 to 3) | 29.21(18.28 to 42.75) | 5(3 to 7) | 31.31(19.30 to 46.37) | 0.24(0.19 to 0.29) |
| **Sao Tome and Principe** | 6(3 to 9) | 52.13(31.42 to 80.43) | 15(9 to 23) | 89.92(54.92 to 138.47) | 1.77(1.75 to 1.79) | 5(4 to 6) | 49.27(38.83 to 62.44) | 12(10 to 16) | 82.32(65.95 to 104.57) | 1.66(1.62 to 1.71) | 0(0 to 1) | 4.92(2.75 to 7.82) | 1(1 to 2) | 8.20(4.88 to 12.54) | 1.66(1.59 to 1.72) |
| **Saudi Arabia** | 986(589 to 1539) | 114.46(68.35 to 178.73) | 4430(2708 to 6777) | 161.32(98.83 to 247.45) | 1.12(1.08 to 1.16) | 1007(828 to 1229) | 128.60(105.22 to 157.28) | 4233(3484 to 5134) | 184.98(151.74 to 225.21) | 1.18(1.13 to 1.23) | 100(62 to 149) | 12.63(7.82 to 18.74) | 426(262 to 636) | 18.15(11.34 to 26.89) | 1.17(1.09 to 1.26) |
| **Senegal** | 182(109 to 283) | 37.13(22.26 to 58.03) | 575(350 to 888) | 49.26(29.98 to 76.11) | 0.91(0.89 to 0.93) | 152(119 to 196) | 35.51(27.82 to 45.84) | 477(378 to 604) | 46.07(36.44 to 58.33) | 0.85(0.78 to 0.92) | 15(8 to 25) | 3.52(1.98 to 5.72) | 48(27 to 76) | 4.56(2.62 to 7.13) | 0.84(0.72 to 0.97) |
| **Serbia** | 1440(855 to 2231) | 76.20(45.34 to 118.65) | 2784(1709 to 4258) | 95.77(58.75 to 146.60) | 0.79(0.66 to 0.93) | 1420(1155 to 1728) | 82.70(66.98 to 101.18) | 2919(2362 to 3591) | 99.32(80.43 to 122.20) | 0.63(0.52 to 0.74) | 143(86 to 216) | 8.26(5.04 to 12.42) | 290(175 to 436) | 9.87(5.93 to 14.84) | 0.61(0.50 to 0.72) |
| **Seychelles** | 4(2 to 5) | 36.76(22.15 to 56.67) | 8(5 to 12) | 38.67(23.65 to 58.58) | 0.18(0.13 to 0.22) | 4(3 to 4) | 37.53(30.38 to 46.40) | 7(6 to 9) | 39.48(32.18 to 48.75) | 0.16(0.12 to 0.21) | 0(0 to 1) | 3.76(2.15 to 5.98) | 1(0 to 1) | 3.93(2.20 to 6.26) | 0.13(0.04 to 0.21) |
| **Sierra Leone** | 111(66 to 173) | 34.38(20.48 to 53.73) | 227(137 to 350) | 40.77(24.56 to 62.96) | 0.54(0.50 to 0.58) | 95(75 to 123) | 32.77(25.78 to 42.39) | 190(150 to 243) | 38.51(30.52 to 49.23) | 0.52(0.49 to 0.55) | 10(5 to 15) | 3.24(1.81 to 5.19) | 19(11 to 31) | 3.82(2.24 to 6.10) | 0.51(0.46 to 0.56) |
| **Singapore** | 327(200 to 493) | 92.95(56.80 to 139.78) | 1566(968 to 2345) | 107.14(66.36 to 160.19) | 0.45(0.36 to 0.54) | 634(525 to 761) | 193.39(159.45 to 232.82) | 2998(2429 to 3686) | 207.50(167.77 to 255.58) | 0.23(0.14 to 0.32) | 64(41 to 93) | 19.38(12.38 to 28.16) | 302(189 to 455) | 20.87(13.04 to 31.37) | 0.24(0.16 to 0.32) |
| **Slovakia** | 2139(1293 to 3332) | 208.15(125.63 to 324.45) | 3082(1916 to 4672) | 186.91(116.21 to 283.56) | -0.24(-0.38 to -0.11) | 2122(1692 to 2662) | 214.86(171.01 to 270.09) | 3095(2489 to 3853) | 188.30(151.11 to 234.66) | -0.38(-0.52 to -0.23) | 210(132 to 313) | 21.20(13.26 to 31.56) | 307(188 to 463) | 18.66(11.44 to 28.12) | -0.35(-0.51 to -0.19) |
| **Slovenia** | 1340(815 to 2072) | 314.15(191.15 to 485.59) | 2344(1468 to 3570) | 283.41(178.06 to 431.66) | -0.30(-0.50 to -0.10) | 1248(970 to 1594) | 302.02(234.77 to 385.55) | 2343(1809 to 3017) | 264.55(205.35 to 339.19) | -0.40(-0.55 to -0.25) | 123(76 to 181) | 29.67(18.46 to 43.65) | 229(140 to 342) | 26.04(15.93 to 39.11) | -0.39(-0.54 to -0.24) |
| **Solomon Islands** | 32(18 to 51) | 79.54(45.68 to 129.33) | 116(68 to 182) | 119.62(70.04 to 189.21) | 1.31(1.27 to 1.36) | 13(10 to 16) | 80.19(62.92 to 101.86) | 49(38 to 62) | 118.76(92.91 to 150.46) | 1.27(1.24 to 1.30) | 1(1 to 2) | 7.90(4.74 to 12.03) | 5(3 to 8) | 11.67(6.96 to 17.76) | 1.26(1.19 to 1.32) |
| **Somalia** | 74(44 to 117) | 23.51(14.02 to 36.98) | 205(125 to 319) | 25.60(15.44 to 39.82) | 0.27(0.25 to 0.30) | 59(47 to 76) | 23.21(18.36 to 29.50) | 158(126 to 201) | 24.96(19.71 to 31.71) | 0.23(0.19 to 0.26) | 6(3 to 10) | 2.30(1.29 to 3.66) | 16(9 to 26) | 2.47(1.37 to 3.99) | 0.22(0.15 to 0.30) |
| **South Africa** | 576(307 to 972) | 17.89(9.53 to 30.21) | 1285(721 to 2103) | 17.43(9.78 to 28.53) | -0.10(-0.14 to -0.06) | 643(515 to 805) | 21.00(16.79 to 26.37) | 1293(1041 to 1629) | 18.98(15.26 to 23.97) | -0.33(-0.40 to -0.27) | 64(40 to 94) | 2.08(1.30 to 3.05) | 128(81 to 189) | 1.86(1.17 to 2.75) | -0.37(-0.46 to -0.28) |
| **South Sudan** | 98(59 to 153) | 25.04(15.01 to 39.02) | 148(89 to 231) | 27.78(16.72 to 43.33) | 0.33(0.31 to 0.36) | 84(66 to 107) | 24.44(19.28 to 31.13) | 120(95 to 153) | 26.56(20.93 to 33.75) | 0.27(0.24 to 0.29) | 8(5 to 14) | 2.38(1.33 to 3.86) | 12(7 to 20) | 2.60(1.46 to 4.19) | 0.27(0.20 to 0.34) |
| **Spain** | 12640(7836 to 18924) | 131.88(81.68 to 197.56) | 30624(18690 to 46026) | 173.77(106.53 to 261.23) | 0.91(0.87 to 0.95) | 25595(20882 to 31354) | 269.04(219.50 to 329.84) | 62050(49817 to 77267) | 331.24(267.98 to 409.03) | 0.68(0.63 to 0.73) | 2544(1619 to 3710) | 26.70(16.99 to 38.93) | 6071(3777 to 8971) | 32.76(20.40 to 48.61) | 0.67(0.57 to 0.77) |
| **Sri Lanka** | 924(561 to 1423) | 56.53(34.26 to 87.48) | 4509(2759 to 6955) | 100.83(61.63 to 155.68) | 1.89(1.85 to 1.93) | 837(680 to 1037) | 57.01(46.25 to 70.80) | 4116(3313 to 5149) | 98.90(79.37 to 124.13) | 1.79(1.76 to 1.83) | 84(50 to 133) | 5.66(3.35 to 8.83) | 407(244 to 619) | 9.68(5.84 to 14.67) | 1.75(1.71 to 1.78) |
| **Sudan** | 323(193 to 506) | 22.41(13.39 to 35.10) | 851(518 to 1317) | 29.45(17.91 to 45.61) | 0.89(0.85 to 0.94) | 369(304 to 449) | 27.88(22.87 to 34.12) | 923(756 to 1132) | 34.41(28.06 to 42.39) | 0.68(0.66 to 0.70) | 37(22 to 58) | 2.77(1.62 to 4.30) | 93(53 to 147) | 3.42(1.97 to 5.38) | 0.68(0.63 to 0.73) |
| **Suriname** | 11(6 to 17) | 26.49(15.76 to 41.98) | 37(23 to 58) | 36.04(21.94 to 56.31) | 0.99(0.96 to 1.02) | 10(8 to 13) | 26.92(21.23 to 34.10) | 36(29 to 46) | 36.15(28.71 to 45.81) | 0.96(0.90 to 1.02) | 1(1 to 2) | 2.70(1.50 to 4.35) | 4(2 to 6) | 3.60(2.05 to 5.66) | 0.93(0.90 to 0.96) |
| **Sweden** | 7978(4295 to 13125) | 292.79(157.84 to 482.61) | 12327(6932 to 19912) | 299.54(168.87 to 482.25) | 0.08(0.04 to 0.12) | 13463(10181 to 17824) | 486.88(370.92 to 639.96) | 20035(15148 to 26388) | 460.25(350.33 to 601.93) | -0.18(-0.25 to -0.11) | 1323(808 to 1995) | 48.00(29.44 to 72.20) | 1944(1190 to 2890) | 45.16(27.69 to 67.21) | -0.19(-0.27 to -0.11) |
| **Switzerland** | 9231(5681 to 13784) | 493.13(303.45 to 736.30) | 13500(8366 to 20132) | 388.80(241.98 to 578.17) | -0.75(-0.84 to -0.67) | 15234(11886 to 19510) | 807.90(634.37 to 1029.60) | 22700(17768 to 29093) | 618.83(486.93 to 786.19) | -0.85(-0.91 to -0.80) | 1488(932 to 2181) | 79.07(49.69 to 115.75) | 2199(1372 to 3220) | 60.64(37.94 to 88.92) | -0.85(-0.91 to -0.79) |
| **Syrian Arab Republic** | 192(114 to 303) | 24.18(14.37 to 38.12) | 708(427 to 1113) | 34.65(20.97 to 54.35) | 1.16(1.06 to 1.26) | 224(184 to 273) | 29.49(24.19 to 36.25) | 756(619 to 927) | 39.53(32.25 to 48.84) | 0.94(0.86 to 1.02) | 22(12 to 36) | 2.94(1.62 to 4.68) | 76(45 to 116) | 3.91(2.33 to 5.97) | 0.94(0.81 to 1.06) |
| **Taiwan (Province of China)** | 2057(1279 to 3124) | 74.30(46.11 to 112.93) | 4256(2638 to 6507) | 56.69(35.13 to 86.67) | -0.87(-0.93 to -0.80) | 1857(1523 to 2285) | 76.80(63.16 to 94.42) | 4142(3319 to 5198) | 54.80(43.88 to 68.81) | -1.08(-1.14 to -1.02) | 190(113 to 289) | 7.77(4.61 to 11.77) | 416(241 to 652) | 5.51(3.18 to 8.65) | -1.09(-1.18 to -1.00) |
| **Tajikistan** | 111(67 to 170) | 23.00(13.97 to 35.38) | 222(135 to 342) | 23.10(14.15 to 35.63) | 0.02(-0.07 to 0.11) | 155(131 to 184) | 34.64(29.11 to 41.02) | 264(219 to 318) | 30.75(25.50 to 37.46) | -0.39(-0.47 to -0.30) | 16(9 to 24) | 3.49(1.99 to 5.40) | 27(15 to 44) | 3.10(1.71 to 5.01) | -0.42(-0.56 to -0.28) |
| **Thailand** | 2447(1468 to 3857) | 44.92(26.89 to 70.86) | 10807(6499 to 16695) | 56.32(33.86 to 87.05) | 0.72(0.68 to 0.77) | 2240(1799 to 2821) | 45.95(36.78 to 58.00) | 11234(9100 to 13948) | 59.53(48.20 to 73.95) | 0.84(0.80 to 0.88) | 225(131 to 355) | 4.57(2.69 to 7.14) | 1120(652 to 1734) | 5.92(3.46 to 9.18) | 0.82(0.75 to 0.90) |
| **Timor-Leste** | 10(6 to 16) | 25.84(15.24 to 40.24) | 61(37 to 94) | 44.65(26.99 to 69.00) | 1.79(1.75 to 1.84) | 9(7 to 11) | 26.77(21.49 to 33.92) | 52(42 to 66) | 42.74(34.25 to 54.23) | 1.54(1.48 to 1.60) | 1(1 to 1) | 2.65(1.52 to 4.21) | 5(3 to 8) | 4.20(2.56 to 6.47) | 1.50(1.43 to 1.57) |
| **Togo** | 70(42 to 109) | 39.74(23.75 to 62.08) | 244(148 to 375) | 45.88(27.77 to 70.60) | 0.46(0.44 to 0.48) | 57(45 to 73) | 37.74(29.84 to 48.22) | 189(150 to 240) | 42.89(33.95 to 54.46) | 0.41(0.39 to 0.43) | 6(3 to 9) | 3.75(2.12 to 5.85) | 19(11 to 31) | 4.26(2.43 to 6.76) | 0.41(0.37 to 0.46) |
| **Tokelau** | 0(0 to 0) | 28.56(16.90 to 44.99) | 0(0 to 0) | 44.41(26.80 to 69.58) | 1.45(1.38 to 1.52) | 0(0 to 0) | 29.46(23.64 to 37.08) | 0(0 to 0) | 43.98(35.22 to 55.26) | 1.29(1.24 to 1.35) | 0(0 to 0) | 2.91(1.66 to 4.61) | 0(0 to 0) | 4.32(2.43 to 6.82) | 1.27(1.18 to 1.37) |
| **Tonga** | 2(1 to 3) | 25.22(14.68 to 40.32) | 4(2 to 6) | 31.30(18.58 to 49.48) | 0.69(0.65 to 0.72) | 2(2 to 2) | 26.93(21.29 to 34.24) | 4(3 to 5) | 32.40(25.67 to 40.88) | 0.59(0.55 to 0.62) | 0(0 to 0) | 2.66(1.44 to 4.37) | 0(0 to 1) | 3.18(1.71 to 5.21) | 0.57(0.51 to 0.62) |
| **Trinidad and Tobago** | 41(25 to 64) | 32.47(19.69 to 50.53) | 87(53 to 133) | 26.01(15.85 to 39.87) | -0.71(-0.77 to -0.65) | 41(33 to 51) | 34.37(27.56 to 42.74) | 97(79 to 119) | 29.37(23.95 to 36.25) | -0.51(-0.56 to -0.45) | 4(2 to 7) | 3.41(1.92 to 5.47) | 10(5 to 15) | 2.92(1.60 to 4.64) | -0.52(-0.62 to -0.42) |
| **Tunisia** | 233(139 to 365) | 29.14(17.34 to 45.62) | 876(527 to 1356) | 40.09(24.10 to 62.09) | 1.04(1.00 to 1.08) | 249(203 to 305) | 34.49(27.91 to 42.67) | 944(767 to 1166) | 45.15(36.55 to 55.95) | 0.89(0.83 to 0.95) | 25(15 to 40) | 3.41(2.01 to 5.37) | 93(55 to 146) | 4.44(2.62 to 6.92) | 0.85(0.78 to 0.91) |
| **Turkey** | 1395(818 to 2245) | 26.49(15.51 to 42.56) | 10110(6045 to 15779) | 67.97(40.58 to 106.09) | 3.11(3.02 to 3.20) | 1503(1208 to 1876) | 30.33(24.28 to 38.10) | 9606(7595 to 12138) | 67.32(53.06 to 85.37) | 2.62(2.53 to 2.71) | 150(83 to 240) | 3.00(1.69 to 4.75) | 948(549 to 1481) | 6.60(3.86 to 10.23) | 2.60(2.42 to 2.77) |
| **Turkmenistan** | 57(34 to 89) | 17.51(10.45 to 27.30) | 104(62 to 166) | 15.72(9.39 to 24.76) | -0.31(-0.42 to -0.20) | 77(65 to 92) | 25.79(21.61 to 30.82) | 139(116 to 168) | 22.28(18.49 to 26.93) | -0.46(-0.51 to -0.42) | 8(4 to 12) | 2.59(1.45 to 4.12) | 14(7 to 23) | 2.24(1.16 to 3.64) | -0.48(-0.56 to -0.40) |
| **Tuvalu** | 0(0 to 0) | 29.20(17.44 to 45.74) | 1(0 to 1) | 43.26(26.20 to 67.70) | 1.28(1.25 to 1.31) | 0(0 to 0) | 29.81(23.66 to 37.82) | 1(1 to 1) | 43.37(34.67 to 54.83) | 1.21(1.18 to 1.23) | 0(0 to 0) | 2.96(1.65 to 4.63) | 0(0 to 0) | 4.30(2.44 to 6.81) | 1.21(1.16 to 1.26) |
| **Uganda** | 297(178 to 466) | 30.97(18.56 to 48.75) | 842(516 to 1303) | 40.91(25.04 to 63.43) | 0.91(0.88 to 0.93) | 246(193 to 318) | 29.80(23.29 to 38.55) | 689(542 to 881) | 38.57(30.20 to 49.26) | 0.82(0.76 to 0.88) | 25(14 to 40) | 2.93(1.64 to 4.65) | 69(40 to 110) | 3.80(2.19 to 6.01) | 0.84(0.80 to 0.87) |
| **Ukraine** | 9805(5249 to 16555) | 78.31(41.86 to 132.24) | 8644(4847 to 14103) | 64.53(36.20 to 105.46) | -0.60(-0.74 to -0.46) | 13928(11585 to 16855) | 113.77(94.57 to 137.85) | 12430(10440 to 14792) | 90.82(76.22 to 108.13) | -0.72(-0.84 to -0.60) | 1384(851 to 2071) | 11.28(6.94 to 16.87) | 1235(783 to 1832) | 9.03(5.71 to 13.40) | -0.72(-0.82 to -0.61) |
| **United Arab Emirates** | 19(11 to 30) | 34.65(20.49 to 54.71) | 247(151 to 379) | 33.10(20.21 to 50.75) | -0.18(-0.22 to -0.13) | 21(17 to 25) | 43.46(35.74 to 52.70) | 271(228 to 320) | 48.25(40.65 to 56.83) | 0.34(0.25 to 0.43) | 2(1 to 3) | 4.33(2.57 to 6.70) | 28(15 to 45) | 4.81(2.72 to 7.59) | 0.35(0.22 to 0.48) |
| **United Kingdom** | 28275(15841 to 45390) | 178.23(99.82 to 286.78) | 53612(31165 to 84086) | 226.67(132.07 to 355.75) | 0.78(0.69 to 0.86) | 52303(41064 to 66649) | 329.02(259.41 to 417.86) | 95620(74808 to 122291) | 390.59(307.16 to 496.15) | 0.57(0.52 to 0.61) | 5167(3264 to 7571) | 32.53(20.58 to 47.54) | 9334(5863 to 13698) | 38.40(24.22 to 56.24) | 0.55(0.50 to 0.59) |
| **United Republic of Tanzania** | 546(326 to 853) | 33.22(19.77 to 51.90) | 1596(969 to 2457) | 43.09(26.13 to 66.35) | 0.84(0.82 to 0.86) | 449(353 to 572) | 32.28(25.29 to 41.38) | 1335(1050 to 1701) | 40.66(31.94 to 51.82) | 0.75(0.70 to 0.80) | 45(24 to 74) | 3.19(1.76 to 5.12) | 134(73 to 213) | 4.03(2.24 to 6.33) | 0.76(0.67 to 0.84) |
| **United States of America** | 101581(54970 to 166077) | 185.40(100.44 to 303.79) | 17(10 to 26) | 306.78(175.06 to 487.12) | 1.67(1.59 to 1.76) | 4(3 to 5) | 326.45(253.43 to 419.16) | 16(13 to 20) | 496.65(383.21 to 636.74) | 1.38(1.31 to 1.45) | 17935(11292 to 26268) | 32.02(20.20 to 46.84) | 2(1 to 2) | 48.04(30.17 to 70.83) | 0.91(0.82 to 1.00) |
| **United States Virgin Islands** | 5(3 to 7) | 38.03(22.60 to 59.34) | 313623(178682 to 497711) | 52.63(32.16 to 81.15) | 1.04(1.01 to 1.08) | 183245(141909 to 235650) | 37.55(29.72 to 47.69) | 527969(407321 to 677167) | 50.24(39.81 to 63.90) | 0.93(0.88 to 0.98) | 0(0 to 1) | 3.75(2.14 to 5.84) | 50901(31981 to 74947) | 4.98(2.85 to 7.74) | 1.33(1.24 to 1.42) |
| **Uruguay** | 820(500 to 1241) | 120.96(73.64 to 183.27) | 1364(832 to 2062) | 135.13(82.63 to 204.15) | 0.35(0.29 to 0.41) | 1445(1165 to 1799) | 216.38(174.36 to 269.72) | 2494(1988 to 3135) | 235.94(189.18 to 294.41) | 0.28(0.22 to 0.35) | 143(90 to 212) | 21.44(13.46 to 31.70) | 244(152 to 364) | 23.29(14.50 to 34.75) | 0.26(0.19 to 0.34) |
| **Uzbekistan** | 401(243 to 617) | 20.29(12.30 to 31.22) | 1067(659 to 1637) | 24.92(15.44 to 38.17) | 0.69(0.60 to 0.78) | 559(475 to 661) | 29.57(25.10 to 34.97) | 1241(1037 to 1505) | 31.62(26.30 to 38.68) | 0.23(0.17 to 0.29) | 57(31 to 90) | 3.00(1.65 to 4.75) | 126(70 to 198) | 3.17(1.79 to 4.96) | 0.20(0.10 to 0.31) |
| **Vanuatu** | 2(1 to 3) | 23.85(14.04 to 37.30) | 8(5 to 12) | 32.10(19.41 to 50.00) | 0.96(0.94 to 0.98) | 2(1 to 2) | 25.20(20.22 to 31.53) | 7(6 to 9) | 32.89(26.31 to 41.31) | 0.86(0.84 to 0.89) | 0(0 to 0) | 2.50(1.40 to 3.98) | 1(0 to 1) | 3.27(1.83 to 5.16) | 0.87(0.82 to 0.92) |
| **Venezuela (Bolivarian Republic of)** | 839(513 to 1291) | 56.97(34.84 to 87.65) | 2304(1387 to 3547) | 46.95(28.29 to 72.16) | -0.62(-0.69 to -0.54) | 834(680 to 1029) | 59.85(48.74 to 74.12) | 2382(1909 to 2982) | 49.15(39.35 to 61.75) | -0.63(-0.69 to -0.58) | 83(49 to 128) | 5.92(3.53 to 9.08) | 237(136 to 371) | 4.88(2.80 to 7.61) | -0.63(-0.72 to -0.53) |
| **Viet Nam** | 4284(2424 to 6951) | 67.10(37.96 to 109.06) | 18537(10963 to 29321) | 122.01(71.94 to 193.42) | 1.96(1.86 to 2.05) | 3707(2881 to 4810) | 63.14(49.04 to 81.94) | 15532(12028 to 20247) | 112.60(86.95 to 147.20) | 1.90(1.84 to 1.96) | 370(215 to 587) | 6.24(3.64 to 9.80) | 1552(916 to 2413) | 11.12(6.61 to 17.15) | 1.90(1.81 to 2.00) |
| **Yemen** | 169(99 to 266) | 22.72(13.32 to 36.02) | 565(338 to 880) | 27.26(16.33 to 42.58) | 0.60(0.55 to 0.64) | 177(145 to 217) | 26.97(21.95 to 33.29) | 586(480 to 720) | 31.21(25.50 to 38.53) | 0.48(0.46 to 0.50) | 18(10 to 28) | 2.68(1.56 to 4.15) | 59(35 to 91) | 3.09(1.85 to 4.73) | 0.47(0.40 to 0.54) |
| **Zambia** | 150(90 to 234) | 36.15(21.71 to 56.25) | 450(273 to 694) | 47.61(28.88 to 73.52) | 0.88(0.83 to 0.93) | 121(96 to 155) | 34.41(27.29 to 43.87) | 358(283 to 458) | 44.74(35.31 to 57.15) | 0.83(0.78 to 0.88) | 12(7 to 20) | 3.39(1.92 to 5.39) | 36(20 to 58) | 4.42(2.55 to 7.00) | 0.86(0.80 to 0.92) |
| **Zimbabwe** | 198(118 to 308) | 33.97(20.11 to 53.01) | 310(189 to 480) | 33.57(20.32 to 52.18) | -0.07(-0.13 to 0.00) | 167(133 to 212) | 33.28(26.32 to 42.88) | 251(200 to 317) | 32.57(25.75 to 41.46) | -0.09(-0.16 to -0.02) | 17(9 to 27) | 3.31(1.86 to 5.31) | 25(14 to 41) | 3.22(1.83 to 5.15) | -0.09(-0.18 to -0.01) |

**Notes:** Rates are reported per 100,000 person-years. Data in parentheses are 95% uncertainty intervals for cases and age-standardized rates of incident, prevalence and YLDs, and 95% confidence intervals for AAPCs. **Abbreviations:** YLDs, years lived with disability; AAPC, average annual percent change; UI, uncertainty interval; ASYR, age-standardized years lived with disability rate; ASPR, age-standardized prevalence rate; ASIR, age-standardized incidence rate.

**Supplemental Table 2.** **Forecast of vertebral fractures caused by falls among elderly people age-standardized Incidence, Prevalence and YLDs rates and cases globally, to 2035.**

|  | **Incident cases** | **ASIR** | **Prevalent cases** | **ASPR** | **YLDs** | **ASYR** |
| --- | --- | --- | --- | --- | --- | --- |
| **Both** | | | | | | |
| **2022** | 2054367(1986241 to 2122492) | 141.02(138.45 to 143.59) | 2727920(2642505 to 2813335) | 193.36(190.04 to 196.68) | 267270(258703 to 275838) | 18.87(18.55 to 19.19) |
| **2023** | 2118526(2030258 to 2206795) | 141.34(137.24 to 145.44) | 2809094(2700510 to 2917677) | 193.31(188.09 to 198.53) | 275056(264233 to 285879) | 18.85(18.35 to 19.36) |
| **2024** | 2191158(2073334 to 2308981) | 141.64(135.62 to 147.66) | 2903153(2760160 to 3046145) | 193.28(185.69 to 200.88) | 284074(269906 to 298242) | 18.84(18.10 to 19.58) |
| **2025** | 2265722(2109899 to 2421544) | 141.90(133.66 to 150.14) | 3000693(2813140 to 3188247) | 193.24(182.92 to 203.56) | 293403(274902 to 311904) | 18.83(17.82 to 19.83) |
| **2026** | 2341802(2139760 to 2543844) | 142.10(131.37 to 152.83) | 3100354(2858338 to 3342369) | 193.07(179.70 to 206.43) | 302924(279123 to 326724) | 18.80(17.50 to 20.10) |
| **2027** | 2420613(2163684 to 2677542) | 142.28(128.79 to 155.77) | 3204039(2897041 to 3511036) | 192.84(176.12 to 209.57) | 312807(282682 to 342932) | 18.77(17.14 to 20.40) |
| **2028** | 2502157(2182259 to 2822055) | 142.45(125.98 to 158.93) | 3313110(2931309 to 3694911) | 192.64(172.30 to 212.98) | 323182(285785 to 360579) | 18.74(16.76 to 20.72) |
| **2029** | 2584040(2193588 to 2974492) | 142.59(122.93 to 162.24) | 3424293(2958437 to 3890150) | 192.44(168.26 to 216.61) | 333735(288177 to 379292) | 18.71(16.36 to 21.06) |
| **2030** | 2665649(2197108 to 3134191) | 142.68(119.65 to 165.70) | 3536690(2977500 to 4095881) | 192.18(163.98 to 220.39) | 344368(289764 to 398973) | 18.67(15.94 to 21.41) |
| **2031** | 2747523(2192861 to 3302186) | 142.70(116.13 to 169.27) | 3650197(2987752 to 4312642) | 191.76(159.34 to 224.18) | 355084(290482 to 419687) | 18.62(15.48 to 21.77) |
| **2032** | 2832734(2182461 to 3483007) | 142.69(112.38 to 173.00) | 3768676(2991180 to 4546173) | 191.22(154.39 to 228.06) | 366240(290513 to 441966) | 18.56(14.99 to 22.13) |
| **2033** | 2922018(2166306 to 3677729) | 142.66(108.43 to 176.89) | 3894325(2989433 to 4799218) | 190.70(149.27 to 232.13) | 378042(290018 to 466065) | 18.50(14.49 to 22.51) |
| **2034** | 3012691(2141834 to 3883547) | 142.58(104.29 to 180.88) | 4022841(2978578 to 5067103) | 190.18(143.99 to 236.37) | 390094(288637 to 491552) | 18.44(13.97 to 22.91) |
| **2035** | 3104585(2108297 to 4100872) | 142.47(99.96 to 184.98) | 4153085(2957059 to 5349110) | 189.62(138.52 to 240.71) | 402288(286218 to 518358) | 18.37(13.43 to 23.32) |
| **Male** | | | | | | |
| **2022** | 791770(759654 to 823886) | 121.29(118.66 to 123.91) | 1053855(1017413 to 1090296) | 173.88(170.68 to 177.08) | 104933(101058 to 108809) | 17.16(16.84 to 17.47) |
| **2023** | 817340(776847 to 857833) | 121.73(117.69 to 125.77) | 1086948(1040874 to 1133021) | 174.08(169.10 to 179.05) | 108177(103355 to 112999) | 17.17(16.67 to 17.67) |
| **2024** | 846519(793694 to 899344) | 122.15(116.32 to 127.99) | 1125363(1065077 to 1185649) | 174.27(167.07 to 181.47) | 111933(105716 to 118150) | 17.18(16.47 to 17.90) |
| **2025** | 876442(807641 to 945244) | 122.54(114.61 to 130.48) | 1164787(1086107 to 1243467) | 174.42(164.65 to 184.19) | 115778(107749 to 123806) | 17.19(16.22 to 18.16) |
| **2026** | 906785(818473 to 995097) | 122.87(112.58 to 133.15) | 1204647(1103462 to 1305832) | 174.44(161.80 to 187.08) | 119670(109419 to 129921) | 17.19(15.93 to 18.44) |
| **2027** | 937715(826301 to 1049130) | 123.15(110.27 to 136.03) | 1245675(1117620 to 1373730) | 174.38(158.57 to 190.19) | 123676(110768 to 136585) | 17.18(15.61 to 18.75) |
| **2028** | 969426(831513 to 1107339) | 123.43(107.73 to 139.13) | 1288724(1129694 to 1447754) | 174.33(155.10 to 193.57) | 127860(111890 to 143829) | 17.17(15.26 to 19.07) |
| **2029** | 1001219(833578 to 1168859) | 123.70(104.99 to 142.41) | 1332499(1138635 to 1526363) | 174.29(151.42 to 197.16) | 132098(112690 to 151506) | 17.16(14.90 to 19.42) |
| **2030** | 1032690(832132 to 1233248) | 123.93(102.02 to 145.83) | 1376255(1143748 to 1608763) | 174.20(147.50 to 200.89) | 136318(113099 to 159536) | 17.14(14.50 to 19.78) |
| **2031** | 1064007(827180 to 1300834) | 124.09(98.81 to 149.36) | 1419876(1144695 to 1695058) | 173.93(143.23 to 204.63) | 140527(113103 to 167952) | 17.11(14.08 to 20.14) |
| **2032** | 1096338(819371 to 1373305) | 124.21(95.38 to 153.03) | 1465037(1142382 to 1787692) | 173.57(138.66 to 208.48) | 144885(112785 to 176986) | 17.07(13.63 to 20.51) |
| **2033** | 1130280(809050 to 1451511) | 124.32(91.77 to 156.87) | 1512943(1137716 to 1888170) | 173.23(133.92 to 212.54) | 149487(112219 to 186756) | 17.03(13.16 to 20.90) |
| **2034** | 1165274(795525 to 1535022) | 124.42(87.99 to 160.85) | 1562206(1129344 to 1995068) | 172.91(129.04 to 216.78) | 154207(111282 to 197131) | 16.99(12.67 to 21.31) |
| **2035** | 1201206(778369 to 1624043) | 124.49(84.03 to 164.96) | 1612141(1116405 to 2107878) | 172.55(123.96 to 221.13) | 158981(109892 to 208070) | 16.95(12.17 to 21.73) |
| **Female** | | | | | | |
| **2022** | 1267678(1228238 to 1307119) | 155.31(152.62 to 158.01) | 1681034(1636402 to 1725667) | 204.97(201.85 to 208.09) | 163171(158064 to 168279) | 19.91(19.58 to 20.23) |
| **2023** | 1306456(1255228 to 1357683) | 155.59(151.27 to 159.91) | 1729990(1671642 to 1788338) | 204.91(199.84 to 209.98) | 167791(161351 to 174231) | 19.88(19.36 to 20.41) |
| **2024** | 1349876(1281338 to 1418414) | 155.82(149.46 to 162.18) | 1786361(1707661 to 1865060) | 204.87(197.38 to 212.37) | 173113(164687 to 181538) | 19.87(19.09 to 20.64) |
| **2025** | 1394494(1303718 to 1485271) | 156.01(147.30 to 164.72) | 1845247(1740353 to 1950140) | 204.83(194.56 to 215.10) | 178662(167659 to 189666) | 19.85(18.80 to 20.90) |
| **2026** | 1440438(1322604 to 1558271) | 156.16(144.82 to 167.51) | 1906028(1769271 to 2042786) | 204.68(191.32 to 218.05) | 184395(170232 to 198558) | 19.82(18.46 to 21.18) |
| **2027** | 1488652(1338610 to 1638695) | 156.34(142.07 to 170.60) | 1970146(1795371 to 2144921) | 204.53(187.74 to 221.33) | 190425(172486 to 208363) | 19.79(18.09 to 21.49) |
| **2028** | 1538651(1351654 to 1725647) | 156.46(139.04 to 173.88) | 2037744(1819253 to 2256236) | 204.40(183.91 to 224.89) | 196763(174484 to 219042) | 19.76(17.69 to 21.83) |
| **2029** | 1588699(1360337 to 1817061) | 156.50(135.73 to 177.28) | 2106570(1839002 to 2374138) | 204.23(179.83 to 228.63) | 203204(176058 to 230351) | 19.73(17.27 to 22.19) |
| **2030** | 1638774(1364626 to 1912922) | 156.49(132.18 to 180.81) | 2176669(1854581 to 2498757) | 204.01(175.50 to 232.52) | 209749(177199 to 242300) | 19.69(16.83 to 22.56) |
| **2031** | 1689603(1364858 to 2014349) | 156.46(128.41 to 184.50) | 2248341(1865820 to 2630862) | 203.65(170.84 to 236.46) | 216439(177898 to 254980) | 19.64(16.35 to 22.94) |
| **2032** | 1742928(1361870 to 2123985) | 156.42(124.43 to 188.41) | 2323927(1873846 to 2774009) | 203.21(165.88 to 240.53) | 223476(178250 to 268702) | 19.59(15.85 to 23.33) |
| **2033** | 1798380(1355288 to 2241471) | 156.31(120.20 to 192.41) | 2403773(1878865 to 2928680) | 202.73(160.70 to 244.76) | 230884(178270 to 283498) | 19.53(15.32 to 23.73) |
| **2034** | 1853831(1343258 to 2364404) | 156.10(115.73 to 196.47) | 2484707(1878098 to 3091315) | 202.21(155.32 to 249.09) | 238377(177708 to 299046) | 19.46(14.78 to 24.14) |
| **2035** | 1909513(1325686 to 2493340) | 155.84(111.07 to 200.60) | 2566720(1871227 to 3262213) | 201.63(149.76 to 253.51) | 245957(176534 to 315380) | 19.39(14.21 to 24.57) |

**Notes:** Rates are reported per 100,000 person-years. Data in parentheses are 95% uncertainty intervals for cases and age-standardized rates of incident, prevalence and YLDs. **Abbreviations:** ASYR, age-standardized years lived with disability rate; ASPR, age-standardized prevalence rate; ASIR, age-standardized incidence rate; YLDs, years lived with disability; UI, uncertainty interval.
