# Supplementary material for: The global burden of vertebral fractures caused by falls among individuals aged 55 and older, 1990 to 2021
Source: PLoS One. 2025 Apr 8;20(4):e0318494. doi: 10.1371/journal.pone.0318494 (PMC11978109; doi:10.1371/journal.pone.0318494)
Supplement: S2 Table — Notes: Rates are reported per 100,000 person-years. Data in parentheses are 95% uncertainty intervals for cases and age-standardized rates of incidence, prevalence and YLDs. ASYR, age-standardized years lived with disability rate; ASPR, age-standardized prevalence rate; ASIR, age-standardized incidence rate; YLDs, years lived with disability; UI, uncertainty interval. (DOCX) [file pone.0318494.s003.docx]

**Supplemental Table 2.** **Forecast of vertebral fractures caused by falls among elderly people age-standardized Incidence, Prevalence and YLDs rates and cases globally, to 2035.**

|  | **Incident cases** | **ASIR** | **Prevalent cases** | **ASPR** | **YLDs** | **ASYR** |
| --- | --- | --- | --- | --- | --- | --- |
| **Both** | | | | | | |
| **2022** | 2054367(1986241 to 2122492) | 141.02(138.45 to 143.59) | 2727920(2642505 to 2813335) | 193.36(190.04 to 196.68) | 267270(258703 to 275838) | 18.87(18.55 to 19.19) |
| **2023** | 2118526(2030258 to 2206795) | 141.34(137.24 to 145.44) | 2809094(2700510 to 2917677) | 193.31(188.09 to 198.53) | 275056(264233 to 285879) | 18.85(18.35 to 19.36) |
| **2024** | 2191158(2073334 to 2308981) | 141.64(135.62 to 147.66) | 2903153(2760160 to 3046145) | 193.28(185.69 to 200.88) | 284074(269906 to 298242) | 18.84(18.10 to 19.58) |
| **2025** | 2265722(2109899 to 2421544) | 141.90(133.66 to 150.14) | 3000693(2813140 to 3188247) | 193.24(182.92 to 203.56) | 293403(274902 to 311904) | 18.83(17.82 to 19.83) |
| **2026** | 2341802(2139760 to 2543844) | 142.10(131.37 to 152.83) | 3100354(2858338 to 3342369) | 193.07(179.70 to 206.43) | 302924(279123 to 326724) | 18.80(17.50 to 20.10) |
| **2027** | 2420613(2163684 to 2677542) | 142.28(128.79 to 155.77) | 3204039(2897041 to 3511036) | 192.84(176.12 to 209.57) | 312807(282682 to 342932) | 18.77(17.14 to 20.40) |
| **2028** | 2502157(2182259 to 2822055) | 142.45(125.98 to 158.93) | 3313110(2931309 to 3694911) | 192.64(172.30 to 212.98) | 323182(285785 to 360579) | 18.74(16.76 to 20.72) |
| **2029** | 2584040(2193588 to 2974492) | 142.59(122.93 to 162.24) | 3424293(2958437 to 3890150) | 192.44(168.26 to 216.61) | 333735(288177 to 379292) | 18.71(16.36 to 21.06) |
| **2030** | 2665649(2197108 to 3134191) | 142.68(119.65 to 165.70) | 3536690(2977500 to 4095881) | 192.18(163.98 to 220.39) | 344368(289764 to 398973) | 18.67(15.94 to 21.41) |
| **2031** | 2747523(2192861 to 3302186) | 142.70(116.13 to 169.27) | 3650197(2987752 to 4312642) | 191.76(159.34 to 224.18) | 355084(290482 to 419687) | 18.62(15.48 to 21.77) |
| **2032** | 2832734(2182461 to 3483007) | 142.69(112.38 to 173.00) | 3768676(2991180 to 4546173) | 191.22(154.39 to 228.06) | 366240(290513 to 441966) | 18.56(14.99 to 22.13) |
| **2033** | 2922018(2166306 to 3677729) | 142.66(108.43 to 176.89) | 3894325(2989433 to 4799218) | 190.70(149.27 to 232.13) | 378042(290018 to 466065) | 18.50(14.49 to 22.51) |
| **2034** | 3012691(2141834 to 3883547) | 142.58(104.29 to 180.88) | 4022841(2978578 to 5067103) | 190.18(143.99 to 236.37) | 390094(288637 to 491552) | 18.44(13.97 to 22.91) |
| **2035** | 3104585(2108297 to 4100872) | 142.47(99.96 to 184.98) | 4153085(2957059 to 5349110) | 189.62(138.52 to 240.71) | 402288(286218 to 518358) | 18.37(13.43 to 23.32) |
| **Male** | | | | | | |
| **2022** | 791770(759654 to 823886) | 121.29(118.66 to 123.91) | 1053855(1017413 to 1090296) | 173.88(170.68 to 177.08) | 104933(101058 to 108809) | 17.16(16.84 to 17.47) |
| **2023** | 817340(776847 to 857833) | 121.73(117.69 to 125.77) | 1086948(1040874 to 1133021) | 174.08(169.10 to 179.05) | 108177(103355 to 112999) | 17.17(16.67 to 17.67) |
| **2024** | 846519(793694 to 899344) | 122.15(116.32 to 127.99) | 1125363(1065077 to 1185649) | 174.27(167.07 to 181.47) | 111933(105716 to 118150) | 17.18(16.47 to 17.90) |
| **2025** | 876442(807641 to 945244) | 122.54(114.61 to 130.48) | 1164787(1086107 to 1243467) | 174.42(164.65 to 184.19) | 115778(107749 to 123806) | 17.19(16.22 to 18.16) |
| **2026** | 906785(818473 to 995097) | 122.87(112.58 to 133.15) | 1204647(1103462 to 1305832) | 174.44(161.80 to 187.08) | 119670(109419 to 129921) | 17.19(15.93 to 18.44) |
| **2027** | 937715(826301 to 1049130) | 123.15(110.27 to 136.03) | 1245675(1117620 to 1373730) | 174.38(158.57 to 190.19) | 123676(110768 to 136585) | 17.18(15.61 to 18.75) |
| **2028** | 969426(831513 to 1107339) | 123.43(107.73 to 139.13) | 1288724(1129694 to 1447754) | 174.33(155.10 to 193.57) | 127860(111890 to 143829) | 17.17(15.26 to 19.07) |
| **2029** | 1001219(833578 to 1168859) | 123.70(104.99 to 142.41) | 1332499(1138635 to 1526363) | 174.29(151.42 to 197.16) | 132098(112690 to 151506) | 17.16(14.90 to 19.42) |
| **2030** | 1032690(832132 to 1233248) | 123.93(102.02 to 145.83) | 1376255(1143748 to 1608763) | 174.20(147.50 to 200.89) | 136318(113099 to 159536) | 17.14(14.50 to 19.78) |
| **2031** | 1064007(827180 to 1300834) | 124.09(98.81 to 149.36) | 1419876(1144695 to 1695058) | 173.93(143.23 to 204.63) | 140527(113103 to 167952) | 17.11(14.08 to 20.14) |
| **2032** | 1096338(819371 to 1373305) | 124.21(95.38 to 153.03) | 1465037(1142382 to 1787692) | 173.57(138.66 to 208.48) | 144885(112785 to 176986) | 17.07(13.63 to 20.51) |
| **2033** | 1130280(809050 to 1451511) | 124.32(91.77 to 156.87) | 1512943(1137716 to 1888170) | 173.23(133.92 to 212.54) | 149487(112219 to 186756) | 17.03(13.16 to 20.90) |
| **2034** | 1165274(795525 to 1535022) | 124.42(87.99 to 160.85) | 1562206(1129344 to 1995068) | 172.91(129.04 to 216.78) | 154207(111282 to 197131) | 16.99(12.67 to 21.31) |
| **2035** | 1201206(778369 to 1624043) | 124.49(84.03 to 164.96) | 1612141(1116405 to 2107878) | 172.55(123.96 to 221.13) | 158981(109892 to 208070) | 16.95(12.17 to 21.73) |
| **Female** | | | | | | |
| **2022** | 1267678(1228238 to 1307119) | 155.31(152.62 to 158.01) | 1681034(1636402 to 1725667) | 204.97(201.85 to 208.09) | 163171(158064 to 168279) | 19.91(19.58 to 20.23) |
| **2023** | 1306456(1255228 to 1357683) | 155.59(151.27 to 159.91) | 1729990(1671642 to 1788338) | 204.91(199.84 to 209.98) | 167791(161351 to 174231) | 19.88(19.36 to 20.41) |
| **2024** | 1349876(1281338 to 1418414) | 155.82(149.46 to 162.18) | 1786361(1707661 to 1865060) | 204.87(197.38 to 212.37) | 173113(164687 to 181538) | 19.87(19.09 to 20.64) |
| **2025** | 1394494(1303718 to 1485271) | 156.01(147.30 to 164.72) | 1845247(1740353 to 1950140) | 204.83(194.56 to 215.10) | 178662(167659 to 189666) | 19.85(18.80 to 20.90) |
| **2026** | 1440438(1322604 to 1558271) | 156.16(144.82 to 167.51) | 1906028(1769271 to 2042786) | 204.68(191.32 to 218.05) | 184395(170232 to 198558) | 19.82(18.46 to 21.18) |
| **2027** | 1488652(1338610 to 1638695) | 156.34(142.07 to 170.60) | 1970146(1795371 to 2144921) | 204.53(187.74 to 221.33) | 190425(172486 to 208363) | 19.79(18.09 to 21.49) |
| **2028** | 1538651(1351654 to 1725647) | 156.46(139.04 to 173.88) | 2037744(1819253 to 2256236) | 204.40(183.91 to 224.89) | 196763(174484 to 219042) | 19.76(17.69 to 21.83) |
| **2029** | 1588699(1360337 to 1817061) | 156.50(135.73 to 177.28) | 2106570(1839002 to 2374138) | 204.23(179.83 to 228.63) | 203204(176058 to 230351) | 19.73(17.27 to 22.19) |
| **2030** | 1638774(1364626 to 1912922) | 156.49(132.18 to 180.81) | 2176669(1854581 to 2498757) | 204.01(175.50 to 232.52) | 209749(177199 to 242300) | 19.69(16.83 to 22.56) |
| **2031** | 1689603(1364858 to 2014349) | 156.46(128.41 to 184.50) | 2248341(1865820 to 2630862) | 203.65(170.84 to 236.46) | 216439(177898 to 254980) | 19.64(16.35 to 22.94) |
| **2032** | 1742928(1361870 to 2123985) | 156.42(124.43 to 188.41) | 2323927(1873846 to 2774009) | 203.21(165.88 to 240.53) | 223476(178250 to 268702) | 19.59(15.85 to 23.33) |
| **2033** | 1798380(1355288 to 2241471) | 156.31(120.20 to 192.41) | 2403773(1878865 to 2928680) | 202.73(160.70 to 244.76) | 230884(178270 to 283498) | 19.53(15.32 to 23.73) |
| **2034** | 1853831(1343258 to 2364404) | 156.10(115.73 to 196.47) | 2484707(1878098 to 3091315) | 202.21(155.32 to 249.09) | 238377(177708 to 299046) | 19.46(14.78 to 24.14) |
| **2035** | 1909513(1325686 to 2493340) | 155.84(111.07 to 200.60) | 2566720(1871227 to 3262213) | 201.63(149.76 to 253.51) | 245957(176534 to 315380) | 19.39(14.21 to 24.57) |

**Notes:** Rates are reported per 100,000 person-years. Data in parentheses are 95% uncertainty intervals for cases and age-standardized rates of incident, prevalence and YLDs. **Abbreviations:** ASYR, age-standardized years lived with disability rate; ASPR, age-standardized prevalence rate; ASIR, age-standardized incidence rate; YLDs, years lived with disability; UI, uncertainty interval.
